# High Stimulus-Related Information in Barrel Cortex Inhibitory Interneurons

**DOI:** 10.1371/journal.pcbi.1004121

**Published:** 2015-06-22

**Authors:** Vicente Reyes-Puerta, Suam Kim, Jyh-Jang Sun, Barbara Imbrosci, Werner Kilb, Heiko J. Luhmann

**Affiliations:** Institute of Physiology, University Medical Center of the Johannes Gutenberg University, Mainz, Germany; UFR Biomédicale de l'Université René Descart, France

## Abstract

The manner in which populations of inhibitory (INH) and excitatory (EXC) neocortical neurons collectively encode stimulus-related information is a fundamental, yet still unresolved question. Here we address this question by simultaneously recording with large-scale multi-electrode arrays (of up to 128 channels) the activity of cell ensembles (of up to 74 neurons) distributed along all layers of 3–4 neighboring cortical columns in the anesthetized adult rat somatosensory barrel cortex in vivo. Using two different whisker stimulus modalities (location and frequency) we show that individual INH neurons – classified as such according to their distinct extracellular spike waveforms – discriminate better between restricted sets of stimuli (≤6 stimulus classes) than EXC neurons in granular and infra-granular layers. We also demonstrate that ensembles of INH cells jointly provide as much information about such stimuli as comparable ensembles containing the ~20% most informative EXC neurons, however presenting less information redundancy – a result which was consistent when applying both theoretical information measurements and linear discriminant analysis classifiers. These results suggest that a consortium of INH neurons dominates the information conveyed to the neocortical network, thereby efficiently processing incoming sensory activity. This conclusion extends our view on the role of the inhibitory system to orchestrate cortical activity.

## Author Summary

Perception of the environment relies on neuronal computation in the cerebral cortex. However, the exact algorithms by which cortical neuronal networks process relevant information from the inputs of sensory organs are only poorly understood. To address this problem we stimulated distinct whiskers and recorded the neuronal responses from identified cortical whisker representations of the rat using multi-site electrodes. For rodents the whisker system is one main sensory input channel, offering the unique property that for each whisker an identified cortical area ("barrel-related column") represents its main cortical input station. In the present study we were able to demonstrate that the action potential firing of single inhibitory neurons provides more information about behaviorally relevant qualities of whisker stimulation (identity of the stimulated whisker and frequency of stimulation) than excitatory neurons. In addition, information about stimulation qualities was encoded with less redundancy in inhibitory neurons. In summary, the results of our study suggest that inhibitory neurons carry substantial information about the sensory environment and can thereby adequately orchestrate neuronal activity in sensory cortices.

## Introduction

A fundamental goal in neuroscience is to understand the complex mechanisms by which populations of neurons process sensory information [1,2]. Substantial progress has been made (1) in estimating the amount of information carried by individual neurons about sensory inputs [3,4], and (2) in determining the importance of functional correlations among neurons for the collective encoding of information [5,6]. Despite these advances, little is known about how specific subpopulations of cortical neurons differ in their encoding capabilities, and the impact of these differences on the processing of sensory information. In this regard, one major focus is to understand the functional impact of cortical inhibitory interneurons in shaping neuronal network activity [7]. Although constituting only ~20% of the total neuronal population, cortical GABAergic interneurons have been implicated in a number of brain functions ranging from the control of neuronal network excitability to higher cognitive processes [8]. In vivo intracellular recordings from different neuronal subpopulations, combined with pharmacological or optogenetic manipulation of GABAergic transmission have started to elucidate the functional role of individual inhibitory cells in different behavioral contexts [9–11]. It is however largely unknown how the activity of interneuron populations contribute to the processing of sensory information in neocortical networks.

In the present study we approached these questions by simultaneously recording with large-scale multi-electrode arrays (of up to 128 channels) the activity of populations of neurons (up to 74) in anesthetized adult rat barrel cortex. Sensory-evoked cortical activity was recorded in response to whisker stimuli which varied in location (up to 3 different whiskers) and frequency (from <1 to 10 Hz, all at physiological range). A systematic approach was employed to segregate the recorded neurons across different putative subpopulations, based on the cortical layers and barrel-related columns in which they were located, and the neuronal type–inhibitory (INH) or excitatory (EXC) [12]. Two different but complementary analyses–mutual information and linear discriminant analysis (LDA)–were subsequently applied to measure the information content in individual and specific subpopulations of neurons [13,14].

With this approach we found a higher level of stimulus-related information in both individual and groups of INH neurons as compared to their EXC counterparts in granular and infra-granular layers, suggesting a sharper tuning of their temporally-precise responses to different stimuli. Moreover, subpopulations of INH neurons presented less information redundancy, which advocates for a more efficient stimulus encoding at the ensemble level when restricted stimulus sets are considered (≤6 stimulus classes). Taken together, our results provide new insights into the mechanisms employed by populations of cortical INH neurons to encode sensory stimuli, and to exert their crucial role in shaping structured network activity.

## Results

**Fig 1:**
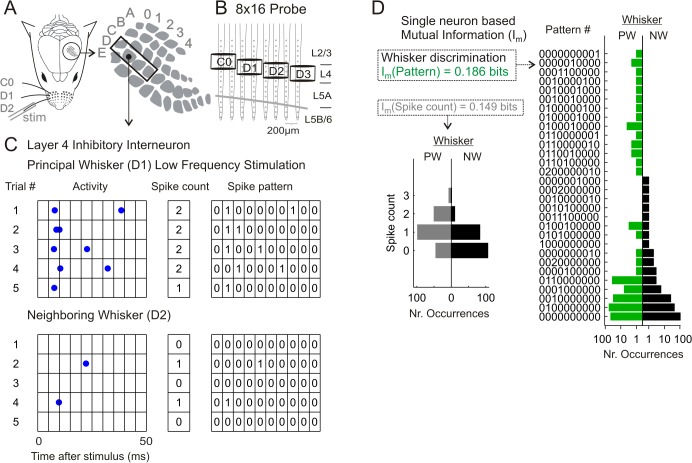
Representation of single neural responses by spike counts and spike patterns. **(A)** Schematic illustration of experimental setup for selective mechanical stimulation of single whiskers and simultaneous VSD imaging or multi-electrode recordings in four barrel-related columns. **(B)** Illustration of the 8x16 channels probe showing shank and site spacing, position of the barrels C0 to D3, and the location of the cortical layers relative to the electrode sites. **(C)** Activity of an inhibitory neuron located at L4 of the barrel D1 in response to principal whisker (PW, upper part) and neighboring whisker (NW, lower part) deflections. Five illustrative trials are plotted for each condition. From the original raster plots (left side), spike counts in a 50 ms time window (middle) and spike pattern vectors of 5 ms bin size (right side) were computed to represent the activity in each single trial. **(D)** Discrimination capacity of the two selected response representations (spike counts and patterns). Left side, distribution of spike counts after PW vs. NW stimulation (200 trials each). Right side, distribution of spike patterns. Note that the overlap of the two distributions is lower for spike patterns than spike counts, thus accounting for higher values of mutual information (I_m_).

Ensembles of well-isolated neurons were recorded with 16- or 128-channel electrode arrays in the barrel cortex of anesthetized adult Wistar rats in vivo. A systematic approach was used in order to allocate the isolated neurons to specific modules of the barrel cortex anatomy [12]. First, barrel-related columns were located by voltage sensitive dye (VSD) imaging upon single-whisker stimulation, which determined the insertion points of the electrode arrays. Local field potential (LFP) responses to stimulation of up to four neighboring single whiskers exhibited a barrel- and layer-specific activation pattern, thus allowing us to allocate the individual recording channels to their respective columns and layers (Fig 1A and 1B). Single neurons were further sorted using a ‘virtual tetrode’ approach, and assigned to the corresponding recording channel containing the maximum negative waveform amplitude (see Methods for further details). Note that no cross-contamination was present among neurons recorded within the same or closely neighboring electrode sites, thus discarding the possibility that the same neural event was assigned to different units (S1 Fig).

A total of 437 neurons from 13 animals are included in the present study. A 1x16 channels probe was used in 4 animals, allowing simultaneous recordings from 5–9 neurons (median 6.5) in a single barrel-related column. In the remaining 9 animals an 8x16 channels probe was employed, allowing simultaneous recordings from 21–74 neurons (median 50) in 3–4 barrel-related columns (median 4). From these 437 neurons, putative inhibitory (INH, 14.2%, n = 62) and excitatory (EXC, 85.8%, n = 375) neurons were identified according to their spike width and waveform asymmetry [15–17], from which 7% (4 INH, 27 EXC) were located in layer 2/3 (L2/3), 14.9% (15 INH, 50 EXC) in L4, 44.6% (14 INH, 181 EXC) in L5A and 33.4% (29 INH, 117 EXC) in L5B/6. In agreement with previous studies [18,19], INH neurons presented distinct spike waveforms, and are associated to higher spontaneous activity than their EXC counterparts across all layers (S1 Fig).

In order to quantify the stimulus-related information conveyed by individual neurons, we represented the neuronal responses using two different measurements, spike counts and spike patterns, for all 263 neurons (recorded from 10 animals) receiving stimulation from the principal whisker (PW) and at least one neighboring whisker (NW) (see below). The intrinsic bias present in the information values (induced by the limited number of trials recorded in each experiment) was corrected in all our analyses using the quadratic extrapolation (QE) correction method (see Methods). Generally, individual neurons responded with a higher number of spikes to principal whisker (PW) than to neighboring whiskers (NWs) stimulation at low frequency (<1 Hz) (Fig 1C), and therefore spike counts already provided information about the stimulus location. However the values of mutual information obtained using spike counts were lower (0.044±0.08 bits) than those obtained using spike patterns (0.11±0.13) (values reported as mean±SD, paired signed-rank test, p<0.001) (Fig 1D, see S2 Fig for extended analyses). The enhanced stimulus discrimination ability provided by the spike patterns derived from the lower overlap between the distributions of responses evoked by either PW or NW stimulation. In agreement with previous reports [3,20], this result confirms that precise spike timing add substantial information about stimulus location.

We next quantified how the window length selected for counting spikes, and the bin size selected to create spike patterns, influenced the level of mutual information about stimulus location within individual cells (S2 Fig). In agreement with previous publications [3,20,21], and taking into account our results collected from the analyses based on LDA classifiers (see below), we selected a 50 ms window length and a 5 ms bin size as appropriate parameters for optimal stimulus discrimination. These parameters were therefore used for further analyses unless otherwise stated.

In addition, we also addressed experimentally the encoding of frequency information. Therefore we applied blocks of stimuli at frequencies ranging from <1 to 10 Hz, using blocks of 200 trials at low frequencies (<1 Hz) and blocks of 100 trials at higher frequencies (from 1 to 10 Hz).

Individual blocks of trials were separated by periods of at least 10 s. As previous reports have shown [22,23], barrel cortex neurons present a decline in the spike count per trial at stimulus frequencies ≥4 Hz. Although some variability could be observed across stimulus frequencies and neuronal groups, neural adaptation occurred typically within the first 10 to 20 first trials (i.e. cycles) within a block, being consistent with previous reports [24]. Thus, at 7 Hz stimulation our neuronal groups showed a significant reduction in their spike counts per trial from the first (0.37±0.24) to the 20^th^ trial (0.17±0.14) (paired signed-rank test, p<0.05). A similar reduction in spike counts was also found at 4 and 10 Hz frequencies (both p<0.05). However, when comparing the spike counts within the 20^th^ and 40^th^ trial, no significant differences could be found at any stimulation frequency (all p≥0.56). This result indicates that stimulus adaptation is a general feature when considering all neuronal groups at 4–10 Hz stimulation frequencies, occurring mainly during the initial stimuli. To exclude the possibility that stimulus adaptation could influence the quantification of information content, we performed all our analyses excluding the first 20 trials from each block containing stimulation frequencies ≥4 Hz.

### Single interneurons in layer 4 convey the highest amount of stimulus location information

**Fig 2:**
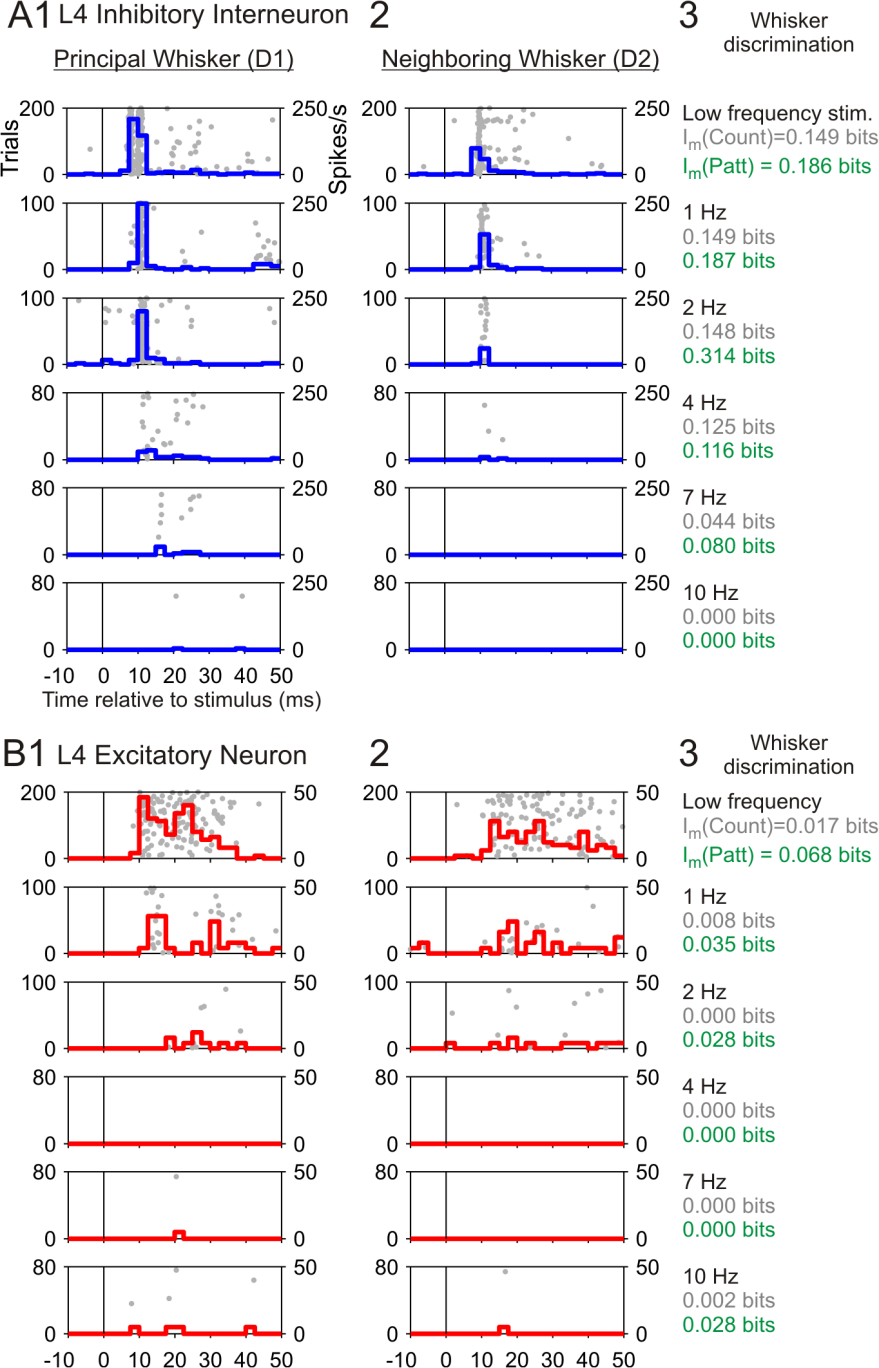
Representative responses of a L4 inhibitory (INH) and excitatory (EXC) neuron located in the same barrel and recorded simultaneously. **(A1)** Responses of the INH neuron to principal whisker (PW) stimulation at different frequencies (same cell as in Fig 1C). Each horizontal subplot represents the activity in response to whisker stimulation at a specific frequency (as indicated in panel A3), containing the raster plot aligned to stimulation and the peri-stimulus time histogram (PSTH). Trials in the raster plot are presented in the same sequential order as recorded. Note that at frequencies ≥4 Hz the first 20 trials (i.e. non-adapted) are omitted. **(A2)** Responses of this INH neuron to neighboring whisker (NW) stimulation, otherwise same as in A1. **(A3)** Values of stimulus location related information for each stimulation frequency, computed using both spike counts (50 ms window length, grey) and spike patterns (5 ms bin size, green). The amount of stimulus frequency related information conveyed by this representative neuron was 0.309 bits for spike counts, and 0.614 for spike patterns. **(B)** Responses of the representative EXC neuron to PW and NW stimulation at different frequencies (same as in A). Regarding frequency related information, this neuron conveyed 0.188 bits when quantified using spike counts, and 0.254 bits when using spike patterns.

We next analyzed whether different neuronal subpopulations show differences in their stimulus decoding capabilities, with special emphasis on the properties of INH and EXC cell populations. First we focused these analyses on L4 neurons, since they are the major cortical target of the lemniscar pathway. At stimulation frequencies ≤4 Hz, L4 INH cells presented relatively high levels of evoked activity after both PW and NW stimulation (Fig 2A1 & 2A2). However, after NW stimulation not only their activity level was lower, but also their first-spike latencies were slightly longer. At higher frequencies the stimulus location information decreased both when the responses were represented as spike counts (50 ms window length) and spike patterns (5 ms time bin) (Fig 2A3).

**Fig 3:**
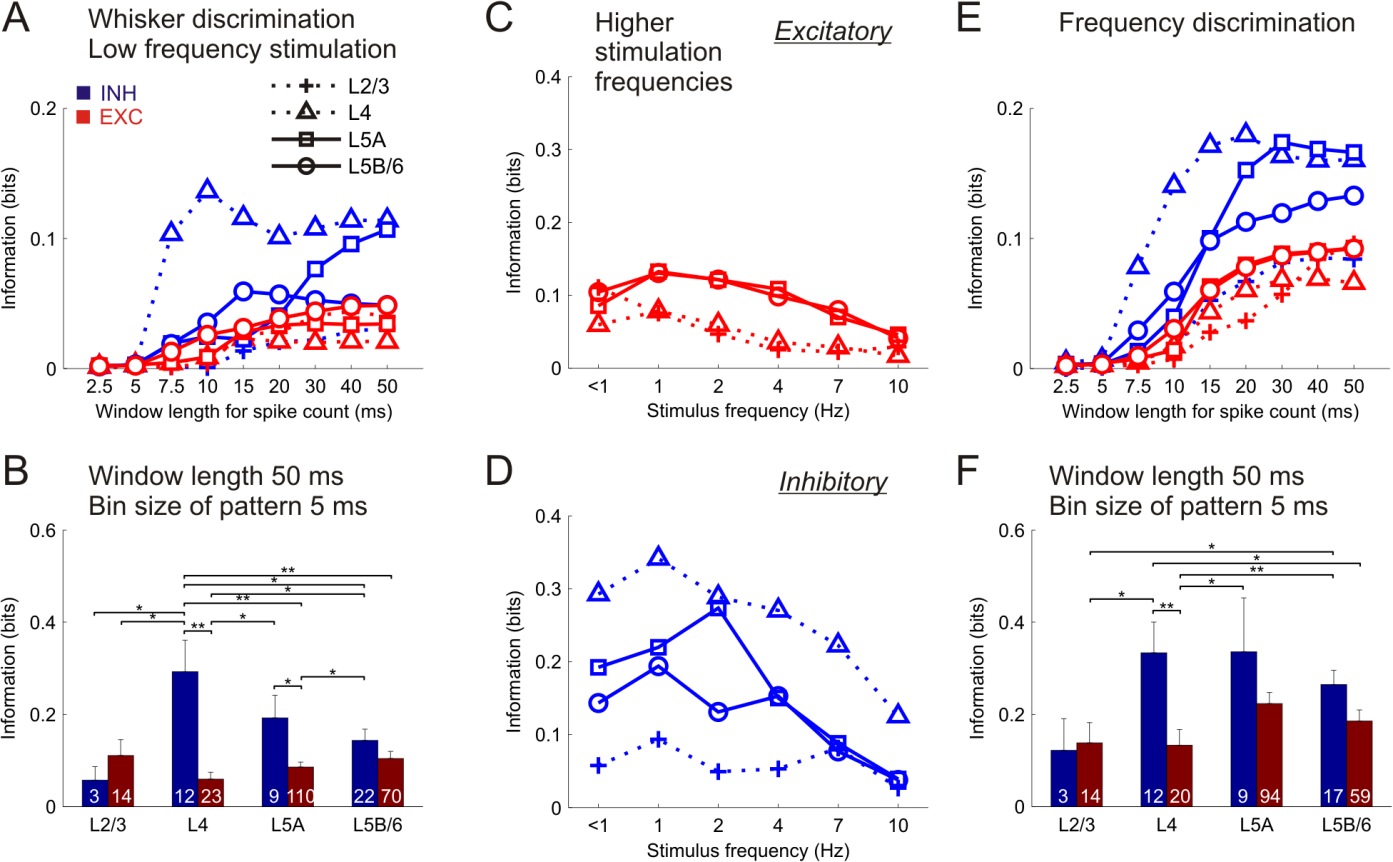
Distribution of stimulus information in single neurons. **(A)** Time course of stimulus location information carried by the spikes counted in increasingly longer time windows for low frequency stimulation (<1 Hz). Data represent the mean values averaged across the specific neuronal subsets (symbols depicted at the legend, subset sizes displayed in panel B). **(B)** Stimulus location information using spike pattern parameters for optimal stimulus discrimination (see S2 and S5 Figs). Each bar represents mean±SEM values. Numbers inside bars represent amount of neurons in each subset. Otherwise same as in A. *P<0.05, **P<0.01. **(C)** Distribution of information at higher stimulation frequencies in different excitatory (EXC) cell groups (symbols represent layers as in A). Data represent the mean values of stimulus location information averaged across the tested stimulation frequencies. **(D)** Distribution of information in inhibitory (INH) cells (same as in C). **(E)** Time course of stimulus frequency information in INH and EXC cells (same as in A). **(F)** Stimulus frequency information using parameters for optimal discrimination (same as in B).

At low stimulation frequencies (≤1 Hz), L4 EXC neurons also revealed a higher level of activity and shorter first-spike latencies in response to PW than to NW stimulation, but their evoked spike responses were less temporally precise than in L4 INH cells (Fig 2B1 & 2B2). Already at frequencies ≥2 Hz the number of evoked spikes for both PW and NW was close to background activity, which resulted in low values for stimulus location information (Fig 2B3). This property together with their lower spike timing precision made EXC neurons less suitable than INH ones for encoding stimulus frequency information (see values of representative neurons in Fig 2 caption, and for extended analyses Fig 3F). These results suggest that L4 INH neurons convey more information about the different stimulus modalities (location and frequency) than L4 EXC neurons, due to (1) their higher number of spikes per stimulus, (2) their higher precision, and (3) their lower adaptation at higher frequency stimulation [24].

Next we compared the mean information conveyed by individual INH and EXC cells segregated across layers. To evaluate the time course of the stimulus discriminability, we first computed the information content of the spike counts in progressively longer time windows (Fig 3A). In this analysis we included those neurons for which their PW and at least one NW were stimulated at low frequency (< 1 Hz) (n = 263 neurons from 10 animals), thus estimating the capability of individual neurons in discriminating PW vs. NW stimulation [3]. L4 INH cells were the fastest and most informative neurons in discriminating the stimulus location, presenting their peak value when the first 10 ms after stimulus were considered (0.14±0.06 bits). L4 INH neurons were also the best to discriminate stimulus location when using the spike counts within an interval of 50 ms after stimulus (0.11±0.04 bits).

Further we quantified the information of the different neuronal groups using optimal parameters for stimulus discrimination (spike patterns of 50 ms length and 5 ms bin size). Except for L2/3, INH neurons carried more information than their EXC counterparts in each layer, with the difference being significant for L4 and L5A (Fig 3B). Remarkably, the amount of information conveyed by L4 INH neurons (0.29±0.07 bits) was significantly higher than that conveyed by all other groups of neurons except L5A INH neurons. The significant outcome of the two-way permutation-based ANOVA test further confirmed the notion that INH neurons convey more information than EXC neurons, since the factor neural type (INH/EXC) was found to be significant (p<0.01).

We next investigated which neural activity parameters could have a major influence on the level of mutual information (computed using a 50 ms time window and 5 ms bin size). In this regard, interneurons are well known to have a relatively high firing rate which can be, under some circumstances, an order of magnitude larger than in pyramidal neurons [18]–a property which could account for the high level of mutual information (see Fig 3B). We found a low but significant correlation between the spontaneous firing rate (FR) and the level of mutual information (R = 0.15, p<0.05), and an even higher correlation between the level of information and the mean number of spikes elicited by PW stimulation (R = 0.55, p<0.001) (low frequency stimulation, n = 263 neurons from 10 animals). Nonetheless and as expected, the highest positive correlation was found between the level of information and the difference between the mean spike number elicited by PW stimulation versus NW stimulation (R = 0.83, p<0.001). Thus, although the level of neuronal activity (in particularly evoked by sensory stimulation) had a significant contribution to the amount of mutual information, the most reliable predictor of the encoding capacity of a neuron was the difference in the level of activity across conditions (PW versus NW stimulation) [3].

The dominance of L4 INH neurons over the remaining neuronal groups in encoding stimulus location persisted at higher stimulation frequencies (up to 10 Hz) (low frequency, n = 263 neurons from 10 animals; higher frequencies, n = 187 neurons from 7 animals) (Fig 3C & 3D). Concordantly, significant differences were found across the neuronal groups at all frequencies tested (permutation test, all p<0.05). Note that in general the neuronal groups presented their highest values at 1–2 Hz stimulation frequencies; at higher stimulation frequencies all neuronal groups showed a gradual decrease in their information content (S3 Fig).

Our data demonstrate that (1) L4 INH neurons are the most important neuronal population for identifying the stimulated whisker at both low and high stimulation frequencies, and (2) barrel cortex neurons encode the location of passive whisker stimuli best at 1–2 Hz stimulation frequencies on a trial-by-trial basis.

### Stimulus frequency is best encoded by single interneurons in granular and infragranular layers

Next we studied which neuronal group provided the highest information about stimulus frequency. In this computation we used only those neurons for which the PW was stimulated at all tested frequencies (< 1, 1, 2, 4, 7 and 10 Hz) (n = 228 neurons from 12 animals). Neural populations showed a parallel increase in their information level when using progressively longer time windows for spike counting, with the peak value for L4 INH cells (0.18±0.04 bits) being at 20 ms after stimulus, and for L5A INH cells (0.17±0.05 bits) at 30 ms after stimulus (Fig 3E).

Further we quantified the stimulus frequency information of single cells as in Fig 3B. L4 and L5A INH neurons carried more information about stimulus frequency than all other groups (0.33±0.07 and 0.34±0.12 bits, respectively) (Fig 3F). In addition, INH neurons conveyed significantly more stimulus frequency information than EXC cells (permutation test, p<0.05). L5A INH neurons were disclosed as best suited for discriminating between different stimulus frequencies, since they presented the highest frequency selectivity in their spike counts (and therefore the highest information under a rate coding scheme) (S4 Fig).

In sum, these results demonstrate that while L4 neurons convey the highest stimulus location information using preferentially temporal coding, both granular and infragranular neurons play a major role for encoding stimulus frequency using a scheme closer to rate coding [25,26]. Our data presented up to this point strongly suggest that INH cells dominate the information content in both granular and infragranular layers.

### Interneuron ensembles encode stimulus location less redundantly than pyramidal cell ensembles

**Fig 4:**
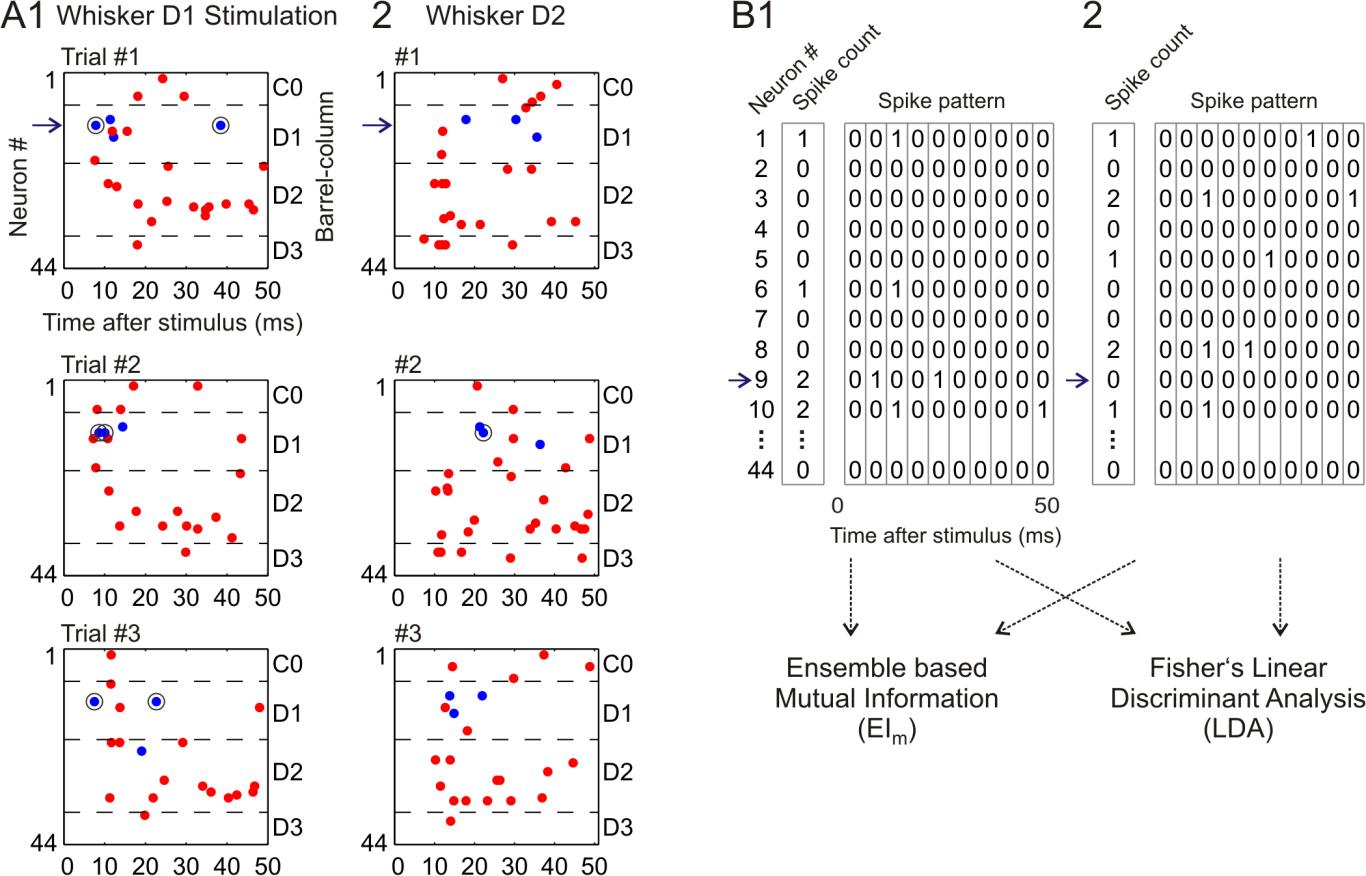
Representation of neural ensemble responses by spike count vectors and spike pattern arrays. **(A1)** Neuronal ensemble activity in response to three trials of whisker D1 stimulation at low frequency. Blue and red dots mark the spike times of INH and EXC neurons, respectively. Cells were ordered along the y-axis according to their position on the shanks, i.e. belonging to the barrel-related columns C0 to D3 (see right side). The numbers at the left side (1 to 44) represent the ordering of the recorded neurons according to their horizontal location. Blue horizontal arrow (together with the black circle around its spike times) marks the INH neuron already presented in Figs 1 and 2A. **(A2)** Neuronal ensemble activity in response to three trials of whisker D2 stimulation (same as in A1). **(B)** Illustration of the method to quantify neural ensemble activity by spike count vectors and spike pattern arrays. Illustrative data are from trial #3 presented in panel A. In general, each trial is represented by a *N*_*neurons*_ spike count vector, or a *N*_*neurons*_ ⋅ *N*_*bins*_ spike pattern array. Afterwards the spike count vectors and spike pattern arrays are used to compute (1) the ensemble-based mutual information, and (2) the decoding performance obtained using linear discriminant analysis classifiers (see text).

Next we expanded our analyses to the neural ensemble level, thereby investigating how populations of neurons jointly represent the studied stimulus modalities, and whether different subgroups of neurons encoded the stimuli synergistically or redundantly. Despite the intrinsic trial-to-trial variability in the evoked population responses, a general propagation pattern could be observed across barrel-related columns (Fig 4A). At low frequency stimulation, neuronal activity started at the principal column ~7 to 10 ms after whisker stimulus, extending toward the first-order neighboring column ~5 ms afterwards [12].

The ensemble responses in each trial were represented as a spike count vector of *N*_*neurons*_ dimensions, or as a spike pattern array of *N*_*neurons*_ ⋅ *N*_*bins*_ dimensions (Fig 4B). We then used the computed spike vectors and arrays to perform two complementary analyses. First, we computed the mutual information provided by the neuronal ensemble, thereby quantifying the contribution of the cross-cell signal and noise correlations on the stimulus-related information. In a second complementary analysis, we constructed LDA classifiers using 75% of the trials and tested the decoding performance with the remaining 25% (four-way cross-validation, see Methods). While the first method quantifies the theoretical discrimination capacity of the two neuronal activity representations [3], the second applies a machine learning algorithm which has been successfully used to predict sensory stimulus properties from neuronal activity on a trial-by-trial basis [13].

First we tested whether the stereotypy in the neural ensemble responses could be used to identify the specific whisker stimulated. To this end, we reduced the dimensionality of the data from the spike pattern arrays by using principal component analysis. The derived principal components formed clusters grouped by the specific whisker stimulated, and could be separated by linear filters, thus supporting the use of LDA classifiers to decode the ensemble responses (S5A Fig). A similar procedure of dimensionality reduction for visualizing the organization of the ensemble responses has been previously described [27]. Further, and in agreement with those results obtained on mutual information at the single-cell level (S2 Fig), the percentage of correct estimates yielded by the LDA classifiers grew as longer time windows for spike counting were used, and thus a window length of 50 ms was used for further analyses (S5B Fig). Interestingly, at higher stimulation frequencies (4–10 Hz) the decoding performance decreased continuously as the temporal resolution of the spike patterns increased (S5C Fig). However, and in order to use homogeneous parameters for all experimental conditions, a bin size of 5 ms (which provided the maximum decoding performance at low frequency stimulation) was established for further analyses.

Next we studied the question whether ensembles of INH or EXC neurons encode stimuli differently. Therefore we created INH and EXC neuronal ensembles of increasing size by compiling neurons from the simultaneously recorded population. When adding neurons in an ‘ascending order’, we selected in each step the neuron conveying lowest information about the stimulus location, quantified using mutual information on individual neurons. On the other hand, we defined as ‘descending order’ the process of selecting firstly those neurons conveying highest information (see Methods).

**Fig 5:**
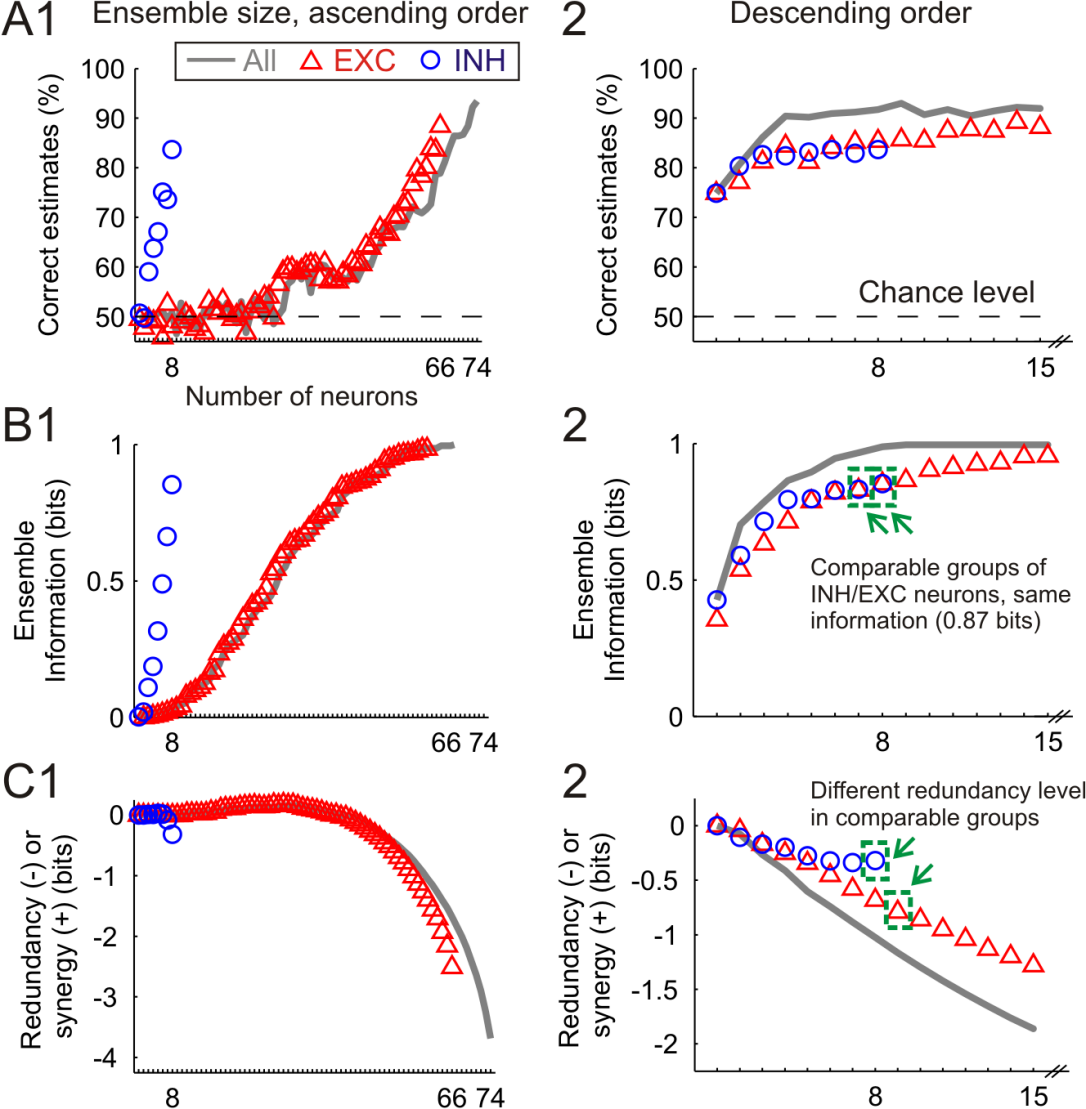
Influence of network size on decoding performance and ensemble-based stimulus location information for a representative experiment. **(A1)** Effect of network size on decoding performance when neurons were chosen in ascending order, i.e. firstly selecting those neurons carrying a lower amount of information (up to n = 74 neurons recorded in this experiment). Three different traces are presented for networks containing only excitatory (red triangles), only inhibitory (blue circles), or both classes of neurons (grey line). Dashed horizontal line represents the chance level (50% since two whiskers were stimulated in this illustrative experiment). **(A2)** Effect of network size on performance when neurons were selected in descending order, i.e. firstly those neurons carrying a higher amount of information. Due to the asymptotic form of the resulting curves, only the values for network sizes of up to 15 neurons are presented in this case. Otherwise same as in A1. **(B1)** Ensemble-based mutual information when neurons are selected in ascending order. The maximum value for the resulting information is 1 bit, i.e. the quantity necessary to distinguish unequivocally between two whiskers. **(B2)** Mutual information when neurons are selected in descending order. Only the values for network sizes of up to 15 neurons are plotted in this case. Green dashed squares represent the highest sized group of INH neurons, and the lowest sized group of EXC neurons carrying at least the same amount of total information than the ensemble of INH cells (0.87 bits, see panel C2). **(C1)** Information redundancy (equivalent to negative values of synergy) in networks selected in ascending order. **(C2)** Information redundancy when neurons are selected in descending order. Note the shorter scale of the y-axis as compared to C1. The redundancy values found in the two equally informative groups (n = 8 INH vs. n = 9 EXC neurons) were directly compared in Fig 6A, along with analogous data from the remaining experiments.

As expected, when applying LDA classifiers to ensembles of neurons selected in ascending order, the decoding performance of both INH and EXC neurons grew supralinearly (Fig 5A1). Remarkably, a small group of INH cells (8 in the illustrative experiment) reached a high decoding performance (83.7%) comparable to a much larger group of EXC cells (65 neurons). This result suggests that while a substantial proportion of EXC neurons conveyed no or little information about the stimulus location, most of INH neurons were highly informative. In contrast, when neurons were selected in descending order, the decoding performance grew promptly and asymptotically for both groups of INH and EXC neurons (Fig 5A2). The 8 most informative EXC neurons presented a decoding performance (85.4%) similar to that reached by INH neurons, indicating that only a small proportion of EXC neurons can be considered as informative as INH neurons, while the majority of EXC cells have a much smaller decoding capacity. Independently of the neuronal type we found that the most informative 9 cells (12.2% of the total) reached a decoding performance (93%) close to that reached by the whole population (93.5%) (Fig 5A2). This finding strongly supports a sparse stimulus encoding scheme in which a minority of cells convey the majority of stimulus-related.

The results obtained by quantifying the ensemble-based mutual information across all experiments further confirmed the previously outlined conclusions. When selecting neurons in an ascending order, smaller groups of 5.3±0.74 INH neurons conveyed as much information (1.01±0.12 bits) as larger groups of low informative EXC cells (28.7±4.2 neurons, 71.7±7.2% of the total) (n = 9 animals) (see illustrative experiment in Fig 5B1). In contrast, relatively small groups containing the most informative EXC neurons (8±2.02 neurons, 21.9±5.6% of the total) reached the same amount of information as the groups of INH cells (Fig 5B2). Further, groups consisting of the 10.1±2.4 most informative INH and EXC neurons (27.7±5.7% of the total) reached the maximum information value.

In order to check whether the most informative EXC neurons (called in the following best EXC neurons) belong to a specific neuronal class, we first tested if the waveforms of the five best EXC neurons in each experiment significantly differed from the remaining EXC neurons (i.e. those less informative) (n = 9 animals). No significant differences were found between the best (n = 45) and the remaining EXC cells (n = 308) either in the spike asymmetry feature (-0.34±0.013 vs. -0.34±0.005, U-test, p = 0.88) or in the spike through to peak latency (0.41±0.007 vs. 0.41±0.002 ms) (independent T-test, p = 0.95), thus indicating that the best EXC cells presented similar spike waveforms as the remaining ones. Further, the best EXC cells presented a laminar distribution similar to that found in all recorded neurons (Chi-Square test, p = 0.92). These data, together with the relatively constant level of stimulus location information found in single EXC neurons across layers (Fig 3B), indicate that the most and least informative EXC neurons are spatially intermingled. Further, no significant differences were obtained in the number of elicited spikes between INH (n = 28) and best EXC (n = 36) neurons, neither when the PW (0.51±0.09 vs. 0.52±0.07) nor when the NW (0.48±0.07 vs. 0.43±0.06) were stimulated (U-test, p = 0.83 and p = 0.4, respectively) (note that for the present comparison we included only INH and best EXC neurons which were stimulated both from their PW and at least one NW). Thus, similar numbers of spikes were elicited by INH and best EXC neurons, and hence their comparable performance–a result confirming the high correlation coefficient found between the level of sensory-evoked activity and the amount of stimulus-related information (see above).

Next we computed the information redundancy (equivalent to negative values of synergy) as the difference between the mutual information reached by the ensemble, and the summed information of all individual cells (computed as if they encoded the stimulus independently, see Methods). When the neurons were selected in ascending order the information redundancy increased exponentially, indicating that the most informative neurons conveyed redundant information (Fig 5C1). Moreover, when the best EXC neurons were directly compared with an equal number of INH neurons, the information redundancy was still higher for the ensemble of EXC neurons (Fig 5C2). Thus, INH neurons could convey as much information as comparable groups including the best EXC neurons, but with less information redundancy, suggesting a more efficient stimulus encoding scheme in INH neurons.

**Fig 6:**
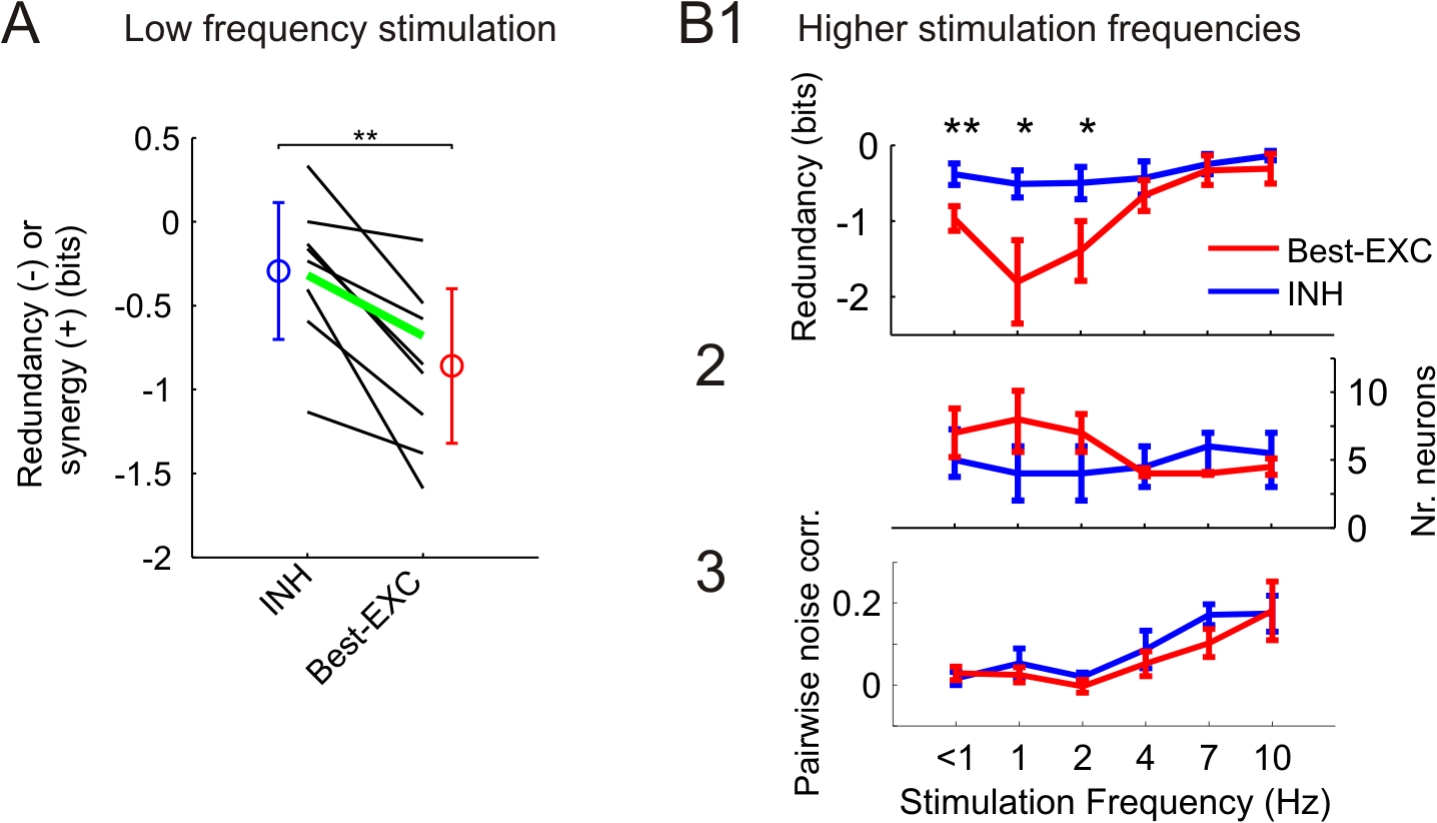
Less redundant (yet independent) encoding of stimulus location by INH neuronal ensembles at different frequencies. **(A)** Comparison of information redundancy present in groups of INH neurons to that in equally informative groups of best EXC neurons at low (<1 Hz) stimulation frequency. Each line represents the corresponding values of an individual experiment (n = 9). Circles and error bars denote mean±SD. *P<0.05. **(B1)** Information redundancy computed similarly as in panel A at different stimulation frequencies. Data represent the mean±SEM averaged across experiments (low frequency, n = 9; higher frequencies, n = 6). *P<0.05, **P<0.01. **(B2)** Number of neurons constituting the groups of INH and best EXC cells. In this case, data represent the median, 40th and 60th percentiles. **(B3)** Level of stimulus-independent noise correlations computed between pairs of cells within the INH and best EXC groups.

In order to test whether this phenomenon was consistent across all recordings, we compared for all experiments (n = 9) the information redundancy present in comparable groups of INH (n = 5.3±0.74) and best EXC (n = 8±2.02) neurons. In this regard, groups of neurons were considered comparable if they conveyed the same amount of ensemble-based information when selected in descending order (1.01±0.12 bits). Groups of INH neurons contained significantly less information redundancy (-0.29±0.13 bits) than comparable groups of best EXC neurons (-0.86±0.15 bits) (paired signed-rank test, p<0.01) (Fig 6A). The lower redundancy in INH cell ensembles was consistently observed at all stimulation frequencies, with the differences being significant up to 2 Hz (Fig 6B1). Note that the groups of best EXC neurons were larger than the groups of INH cells at stimulation frequencies ≤2 Hz (Fig 6B2). In addition, the mean amount of information contained in individual neurons was slightly higher in individual EXC than INH neurons at all stimulation frequencies (not significantly, paired signed-rank test, all p≥0.16, S1 Dataset). As a consequence, the higher number of cells included in the best EXC groups, carrying no lower amount of information than INH cells individually, suggested a larger overlap in their information content (a feature which was quantified as a higher level of redundancy).

We further evaluated this hypothesis by applying the information breakdown methodology in comparable groups of INH (n = 5.3±0.74) and best EXC (n = 8±2.02) neurons (Table 1). For this computation, neural ensemble responses were quantified using spike counts in a post-stimulus window of 50 ms, which allowed us (1) to keep the computational costs within a feasible level, and (2) to directly compare the resulting values to those obtained by computing pairwise noise correlations (see below). At low frequency stimulation, best EXC cell groups presented a significant bias toward more negative values in the signal-similarity term (-0.23±0.06) when compared to INH cell groups (-0.09±0.03 bits) (n = 9 animals, paired signed-rank test, p<0.05), indicating that best EXC cell mean responses tended to be better correlated and therefore more similar, while INH neurons tended to be more variable. Note that the differences in the signal similarity term between INH and best EXC neurons were significant at all stimulation frequencies except 4 Hz (all p<0.05, Table 1). Moreover, the noise correlation term was mainly dominated by the stimulus-dependent factor, which was not significantly different between both cell groups at any stimulation frequency (all p>0.3, S1 Dataset). Further, the contribution of the stimulus-independent noise correlations was small (as compared to the signal similarity term) in both INH and best EXC cell groups at all stimulation frequencies, and not significantly different from each other (all p>0.06, Table 1).

**Table 1:** Information breakdown methodology applied to groups of INH vs. best EXC (B-EXC) neurons.

Term		< 1 Hz (n = 9)	1 Hz (n = 6)	2 Hz (n = 6)	4 Hz (n = 6)	7 Hz (n = 6)	10 Hz (n = 6)
Sig-sim	INH	-0.09±0.03	-0.14±0.06	-0.22±0.1	-0.31±0.16	-0.08±0.05	-0.03±0.01
	B-EXC	-0.23±0.06	-0.63±0.2	-0.68±0.18	-0.39±0.1	-0.2±0.07	-0.07±0.04
	*P*	* <0.*05*	* <0.*05*	* <0.*05*	0.56	* <0.*05*	* <0.*05*
Corr-ind	INH	-0.01±0.01	-0.01±0.02	-0.01±0.02	-0.05±0.02	-0.02±0.01	-0.01±0.01
	B-EXC	-0.02±0.02	-0.04±0.02	-0.02±0.01	-0.02±0.02	-0.05±0.02	-0.07±0.03
	*P*	0.10	0.06	0.56	0.31	0.31	0.09

Neural ensemble responses were quantified using spike counts in a post-stimulus window of 50 ms. Two components of the information breakdown were computed and compared: the signal-similarity term (sig-sim) and the stimulus-independent correlational component (corr-ind). Data represent the mean±SEM averaged across experiments (low frequency, n = 9; higher frequencies, n = 6). The number of neurons included in each of the INH and best EXC neuronal groups are as reported in Fig 6B2. The corresponding pairwise noise correlations are shown in Fig 6B3. Significant differences are highlighted in cursive and marked with an asterisk.

This result was further corroborated by computing the level of stimulus-independent noise correlations for each cell pair–i.e. the Pearson correlation of the spike counts of pairs of neurons in response to presentations of the same stimulus (50 ms time window). Stimulus-independent noise correlations were close to 0 at lower stimulation frequencies (≤2 Hz) and moderate (~0.2) at higher stimulation frequencies (7–10 Hz) (Fig 6B3), with no significant differences between groups of INH and best EXC neurons (paired signed-rank test, all p≥0.16). In sum, these results demonstrate that (1) the activity of cell pairs was mainly decorrelated at all frequencies independently of the neuronal type, suggesting that the stimulus location was encoded independently by the neurons participating in the ensembles, and thus (2) the lower information redundancy observed in ensembles of INH neurons likely arose from the higher variability in their mean responses.

The differences in redundancy within ensembles of INH and best EXC neurons were not due to a lower level of anatomical dispersion of the best EXC neurons, i.e. being located more often in the same barrel-related columns or layers [5]. No significant differences were found between INH and best EXC cell groups either in the total number of columns in which they were scattered (paired signed-rank test, all p≥0.25), or in the entropy level of the cell distribution across columns (all p≥0.31). Similar results were obtained in relation to the cell distribution across layers (see above). These data indicate that INH and best EXC neurons were equally dispersed across the channels in our recording probes, and thus across the barrel cortex anatomy.

When comparing the results from the LDA classifiers to the mutual information, a proper control analysis is to quantify the LDA performance using the transmitted information (i.e. the mutual information of the confusion matrix between real and decoded stimulus class). At low frequency stimulation the stimulus location information of INH neurons was higher when computed using the direct (debiased) method (1.01±0.12 bits) than when computed using the transmitted information quantification (0.46±0.06 bits) (paired signed-rank test, p<0.01). An increase from transmitted to direct information values was also obtained when considering best EXC neurons (from 0.62±0.07 to 1.08±0.1 bits, p<0.01), and all neurons within the population (from 0.85±0.07 to 1.18±0.1 bits, p<0.01). Further, analogous increases were achieved when computing the stimulus location information at higher frequencies, or when considering another stimulus modality–namely the stimulus frequency information (S1 Dataset). These results are in agreement with previous publications, in which the transmitted information has been proposed as a lower bound for the true information conveyed by the network [28].

**Fig 7:**
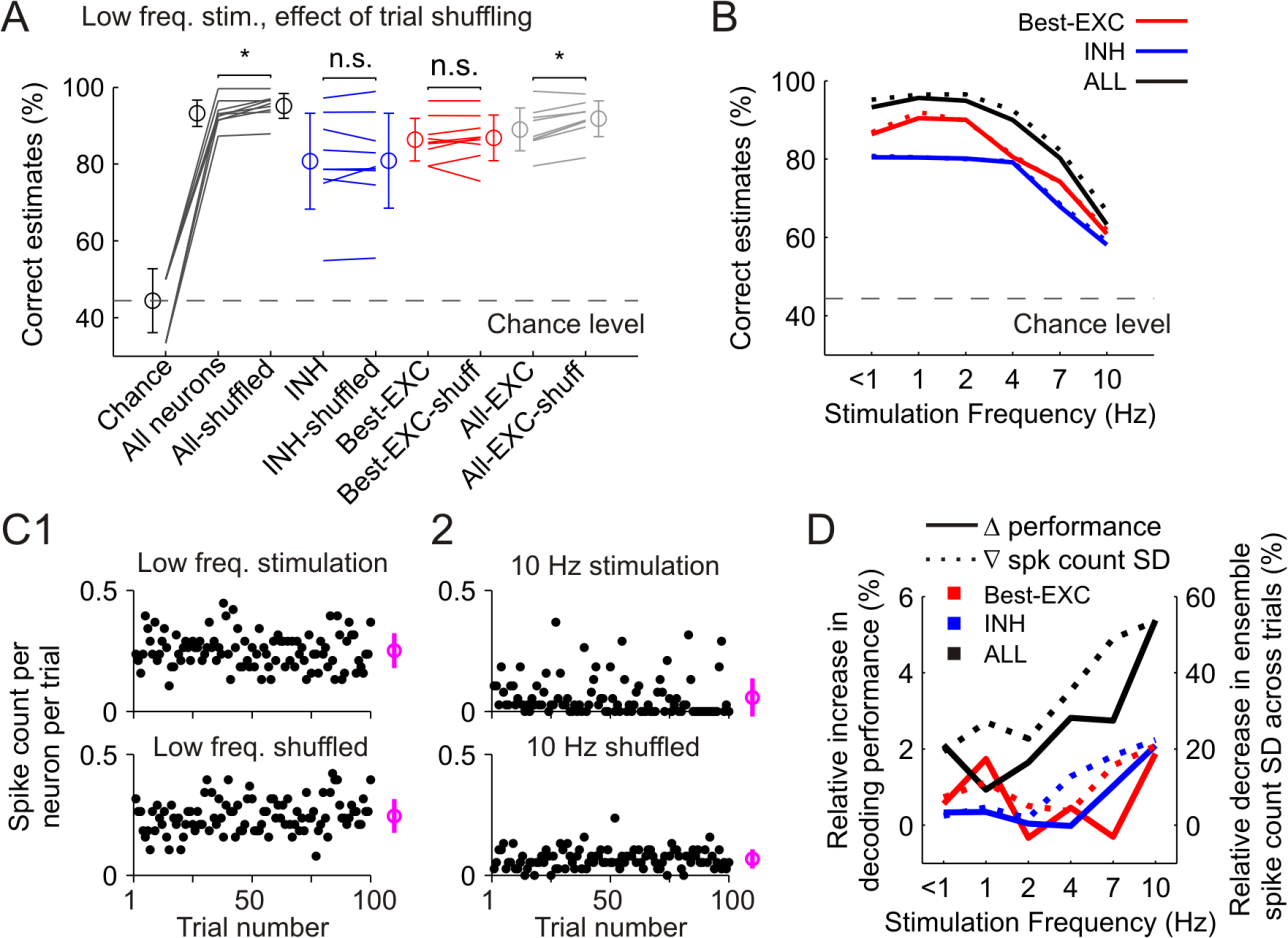
Performance of linear discriminant analysis (LDA) classifiers for decoding stimulus location at different frequencies. **(A)** Impact of trial-shuffling on performance of the LDA classifiers at low frequency stimulation. From left to right, performance reached by networks containing the total amount of neurons in the recording (black), only INH neurons (blue), only the best EXC neurons (red), or all EXC neurons (grey). Circles and error bars denote mean±SD. Dashed line marks mean chance level averaged across all experiments (44.4%, n = 9). *P<0.05. **(B)** Decoding performance at higher stimulation frequencies using all neurons (black), only the INH ones (blue) or the best EXC ones (red). Continuous lines represent the mean decoding performance averaged across experiments. Thick dashed lines represent decoding performance after trial-shuffling. **(C1)** Upper part, progression of the ensemble activity during 100 trials of stimulation at low frequency in a representative experiment. Dots represent the number of spikes elicited per neuron (y-axis) in a specific trial (x-axis). Circle and bar at the right side represent mean±SD averaged across all trials. Lower part, surrogate data generated by trial-shuffling from the original activity presented in the upper part. **(C2)** Upper part, progression of the ensemble activity at 10 Hz whisker stimulation. Note that the first 20 trials (i.e. non-adapted) are omitted. Lower part, surrogate data generated by trial-shuffling. Note the more homogeneous distribution of the activity across trials. **(D)** Comparison of the relative increase in decoding performance obtained after trial-shuffling to the decrease in the variability of the ensemble activity level averaged across trials. Data are scattered across stimulation frequencies and neuronal groups.

To further test the level of independence with which populations of barrel cortex neurons encode stimuli, we analyzed the performance of the LDA classifiers applied on surrogate population responses generated by trial-shuffling (see Methods). After such computational manipulation, which destroys noise but not signal correlations, neurons can be considered as representing the stimulus independently from each other–i.e. independently in the sense that the activity of one cell does not influence the activity of the other (for instance through common connectivity) [3,21]. At low frequency stimulation (< 1 Hz) we found no effect of trial-shuffling when only INH (p = 0.84) or best EXC neurons (p = 0.57) were considered (Fig 7A) (paired signed-rank test). However, when all neurons or all EXC neurons were used as a population, the performance of the decoders was significantly improved after trial-shuffling (paired signed-rank test, both p<0.05) [21,29,30]. At higher stimulation frequencies the proportion of correct estimates gradually decreased (Fig 7B, see also Fig 3C & 3D); however, the relative increases in decoding performance after trial-shuffling grew as higher stimulation frequencies were used. For groups including all cells, the relative increase in decoding performance grew from 2.1% at low frequency stimulation, to 5.4% at 10 Hz (see below Fig 7D). These results indicate a neutral or positive net effect of trial-shuffling on decoding performance, with this effect being more beneficial when cross-cell noise-correlations were higher (i.e. at higher stimulation frequencies, see Fig 6B3).

We next investigated the cause for the increase in stimulus decoding performance after trial-shuffling at higher stimulation frequencies. Typically, at low frequency stimulation (<1 Hz) the number of spikes elicited per neuron was rather stable across trials (Fig 7C1, upper part). Thus, the variability of the ensemble responses across trials, measured as the SD of the spike count per neuron, was similar in real and trial-shuffled responses (0.07 spikes) (Fig 7C1, lower part). However, at higher stimulation frequencies (10 Hz) the variability of the real ensemble responses per trial was higher (0.08 spikes), since some trials contained a high number of spikes per neuron, while the majority contained very few (Fig 7C2, upper part). In this case, trial-shuffling redistributed the few spikes elicited per neuron, so that the number of spikes was homogeneously distributed across trials, thereby reducing the variability in the level of the ensemble responses (0.04 spikes) (Fig 7C2, lower part). As a consequence of this reduction in the variability of the responses, the stimulus decoding performance was enhanced. Thus, the relative decrease in the ensemble spike count variability after trial-shuffling was correlated to the relative increase in decoding performance across individual experiments and stimulation frequencies (Fig 7D). This correlation was significant when all established cell groups were considered together (low frequency, n = 9 animals; higher frequencies, n = 6; Pearson correlation, R = 0.36, p<0.001)–thereby demonstrating that when a limited number of spikes are available per trial, it is beneficial for the decoding performance to reduce the variance of the activity across trials, which in turn is translated into lower levels of stimulus-independent noise correlations between neurons.

As elaborated in Fig 3E and 3F and related text, individual neurons conveyed information not only about the stimulus location, but also about the frequency used for stimulation. Next we examined how neuronal ensembles jointly represent stimulus frequency, and whether the same encoding strategy is used as for stimulus location. In order to address these questions, we first varied the window length and bin size used for stimulus frequency decoding, obtaining results in line with those observed for stimulus location (S6 Fig).

**Fig 8:**
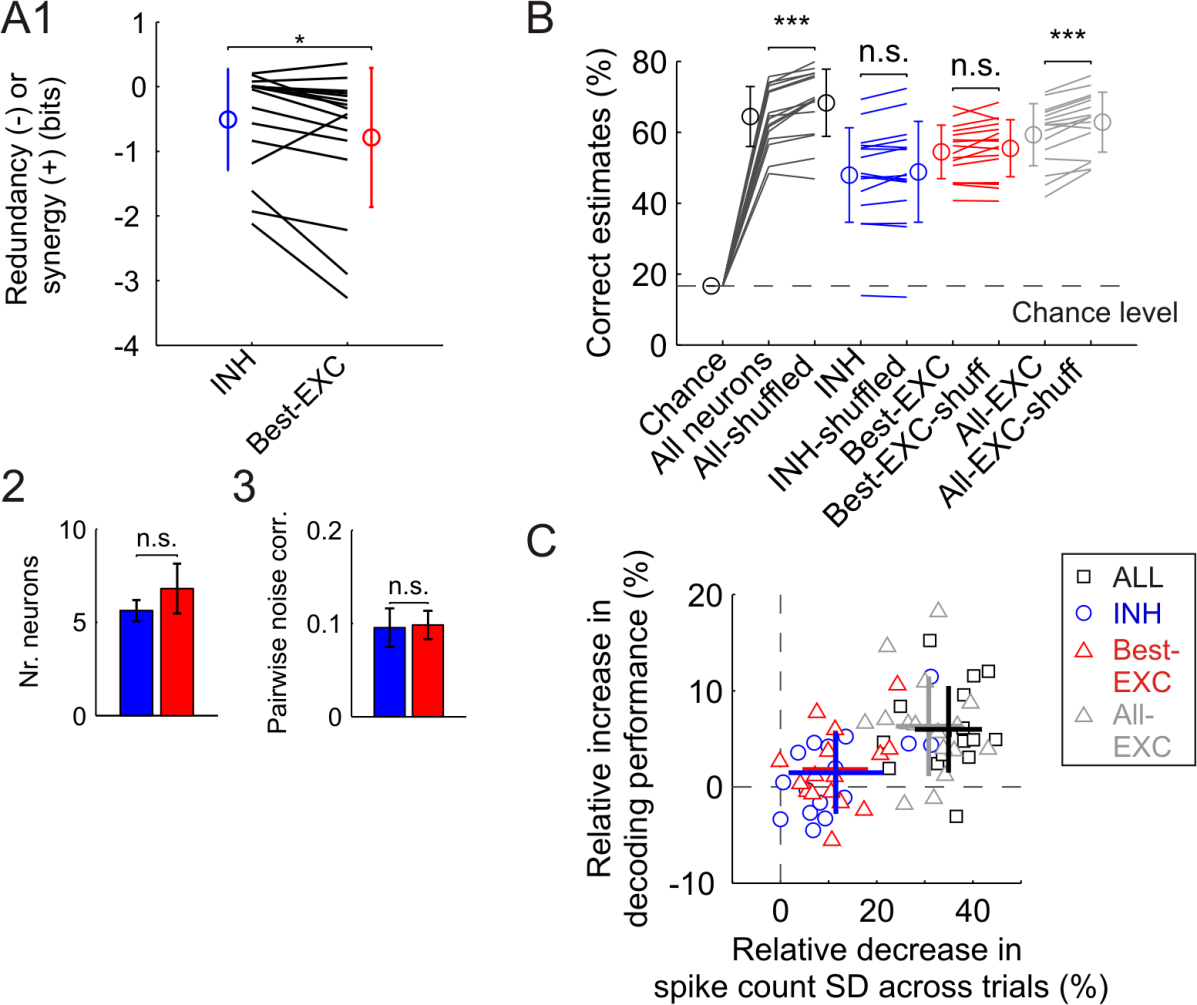
More efficient encoding of stimulus frequency by INH neuronal ensembles. **(A1)** Comparison of information redundancy present in groups of INH neurons to that in equally informative groups of best EXC neurons. Each line represents the corresponding values of an individual dataset, including the stimulation blocks at all tested frequencies for a specific whisker and animal (n = 16 datasets from 8 animals). Circles and error bars denote mean±SD. *P<0.05. **(A2)** Number of neurons constituting the groups of INH and best EXC cells. Bars and error bars denote mean±SEM. **(A3)** Level of stimulus-independent noise correlations averaged across datasets. **(B)** Impact of trial-shuffling on decoding performance of the liner discriminant analysis decoders (same as in Fig 7A). ***P<0.001. **(C)** For each neuronal group, comparison of the relative increase obtained in decoding performance after trial-shuffling to the decrease in the variability of the ensemble activity level averaged across trials. Crosses represent mean±SD of the computed values for each neuronal group.

We further computed the amount of redundancy related to stimulus frequency information. As previously, we compared groups of INH (n = 5.6±0.56) and EXC (n = 6.8±1.3) neurons selected in descending order and conveying an equal amount of ensemble information (1.69±0.18 bits). Groups of INH neurons contained significantly less information redundancy (-0.51±0.2 bits) than comparable groups of best EXC neurons (-0.79±0.27 bits) (n = 16 datasets from 8 animals, paired signed-rank test, p<0.05) (Fig 8A1). Since best EXC neurons carried stimulus frequency information more redundantly, a slightly higher number of neurons were needed in the best EXC groups (6.8±1.3) to reach similar levels of ensemble-based information than in the INH groups (5.6±0.56) (n = 16 datasets, paired signed-rank test, p = 0.27) (Fig 8A2). This result suggests that the property of INH neurons encoding stimuli more efficiently than EXC neurons is not specific to one stimulus modality (location), but rather a general phenomenon.

The results derived from the information breakdown methodology revealed trends similar as for stimulus location: (1) mean signals were more similar within best EXC cell groups than within INH cell groups, and (2) noise correlations were dominated by the stimulus-dependent factor (S1 Dataset). Accordingly, the level of the stimulus-independent noise correlations was equal in INH (0.096±0.02 bits) and best EXC (0.098±0.02 bits) cell pairs (paired signed-rank test, p = 0.76) (Fig 8A3). Note that the moderate cross-cell correlations obtained here were influenced by those observed at higher stimulation frequencies (see Fig 6B3). This result thus corroborates that stimulus frequency (as well as stimulus location) was encoded rather independently by individual neurons, and that the stimulus-independent noise correlations did not play a significant role in stimulus encoding.

We next tested the performance of the LDA classifiers using trial-shuffled population responses. No effect of trial-shuffling was found when only INH (n = 16 datasets from 8 animals, paired signed-rank test, p = 0.17) or the best EXC neurons (p = 0.11) were considered (Fig 8B). However, when all neurons or all EXC neurons were used as a population, the performance of the decoders was significantly improved after trial-shuffling (paired signed-rank test, both p<0.001) [21,29,30].

As when decoding stimulus location, a relationship was observed between the relative decrease in the ensemble spike count variability and the increase in decoding performance after trial-shuffling (Fig 8C). The correlation between these two factors was significant when the four established cell groups were considered together (n = 16 datasets from 8 animals, values compared by dataset, Pearson correlation, R = 0.5, p<0.001). Thus, the beneficial contribution of trial-shuffling to decoding performance is higher when the activity level within the original (i.e. real) ensemble responses is less homogeneously distributed across trials–a property in turn induced by higher values of cross-cell stimulus-independent correlations.

Taken together, our results demonstrate that (1) the observed stereotypy in the population responses can be used to decode both stimulus features (location and frequency) in a trial-by-trial basis, (2) INH neurons convey as much stimulus-related information as the ~75% least informative EXC neurons, and as much as the ~20% most informative ones, (3) INH neurons present a lower amount of information redundancy than comparable groups of best EXC neurons, and (4) stimulus-independent cross-cell noise correlations were however low within both INH and EXC cell groups, and therefore their responses could be considered to be independent of each other without detriment (but rather improvement) in the decoding performance.

## Discussion

**Fig 9:**
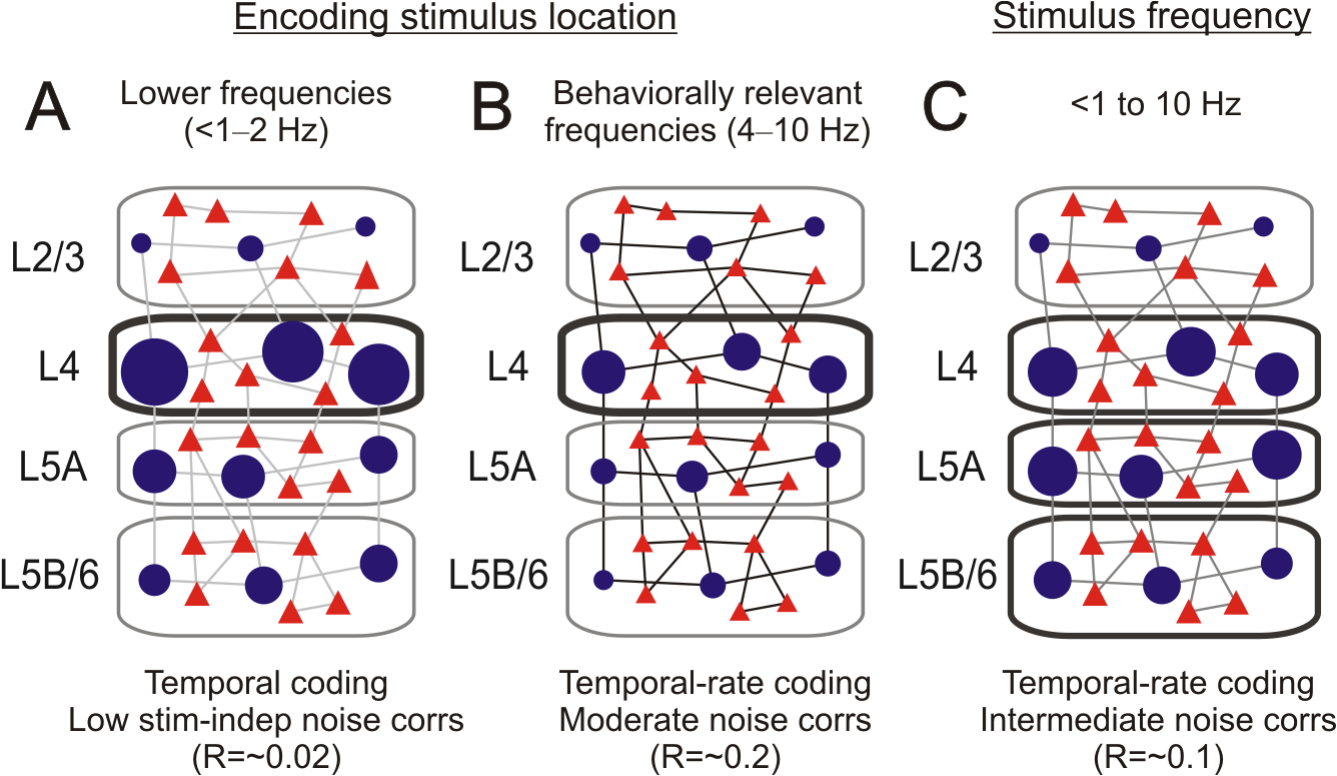
Summary diagram of the encoding schemes for the different stimulus modalities. **(A)** Scheme proposed for encoding stimulus location at lower stimulation frequencies (≤2 Hz). Symbols illustrate the INH (blue circles) and EXC (red triangles) neurons inside the principal column of the stimulated whisker. Size of the symbols indicates the amount of stimulus location information carried by the corresponding neuronal groups (i.e. their capability in discriminating principal vs. neighboring whisker stimulation), distributed across the cortical layers. Lines connecting the neurons mark the level of the stimulus-independent noise correlations, where lighter tones of gray relate to lower values. Noise correlations between INH and EXC cells are not represented. Other properties as the proportion of cell types per layer, the level of spontaneous firing rate or the number of synaptic connections between neurons are not taken into consideration. See text for further details. **(B)** Encoding regime of stimulus location at behaviorally relevant stimulation frequencies (4–10 Hz). Otherwise same as in A. **(C)** Scheme proposed for encoding stimulus frequency in the principal column of the stimulated whisker.

In the present study we investigated the encoding capabilities of subpopulations of neurons in the barrel cortex in vivo, with a special emphasis on the differences between INH and EXC neuronal ensembles. Our data revealed different encoding regimes employed by these neuronal types in order to represent the applied stimulus parameters (location and frequency). At lower stimulation frequencies, L4 INH neurons carried the highest amount of stimulus location related information, with spike timing being crucial for stimulus encoding (Fig 9A) [3,31]. Noise correlations were very low among neurons of all types, indicating that the activity of each individual neuron was mainly decorrelated from the remaining ones [5,29]. At behaviorally relevant stimulation frequencies (4–10 Hz) a similar distribution of information was found; however, the information was lower in all neuronal groups, and the network employed a encoding scheme closer to rate coding (Fig 9B) [25]. In this case noise correlations were moderate, a property which is detrimental for the resulting trial-by-trial decoding performance [21,29,30]. For encoding stimulus frequency, INH neurons in granular and infragranular layers [25] were the most informative ones (Fig 9C). Noise correlations were in this case intermediate, which was detrimental for the encoding capacity of the least informative EXC neurons, but not the INH or the most informative EXC neurons.

Remarkably, all encoding regimes were dominated by the higher information conveyed by INH cells, which carried as much information as the ~20% most informative EXC cells, and the ~75% less informative ones. Although in the cortex the number of EXC neurons is about an order of magnitude larger, the majority of these neurons conveyed rather little amount of information about the tested stimulus sets. These findings agree with recent studies performed in the auditory cortex of awake macaques, in which a small fraction of temporally precise neurons carried the majority of information about natural sounds [32]. Taken together, these data strongly support a sparse stimulus encoding scheme for EXC cells, in which a minority of cells convey the majority of stimulus-related information. However, in the present study we found this sparseness to be less pronounced in INH cells, which present a more homogeneous distribution of information supported by their higher temporal precision. Further, these data suggest that a rather similar amount of information might be represented by the total population of EXC neurons as compared to INH neurons, however with a higher degree of redundancy, which might be important for the robustness and propagation of sensory information (see below).

We propose the high stimulus selectivity to be a salient property of barrel cortex INH neurons, which might serve to efficiently shape structured cortical activity [8,33]. Before further discussing the present data, it is important to note that the INH neurons–classified as such based on specific extracellular spike features [12]–correspond to a large extent to the fast-spiking interneuron subpopulation [7,9,10]. Future studies combining extracellular recordings with labeling/imaging techniques should further evaluate the function of the heterogeneous non fast-spiking GABAergic interneuron subtypes at the population level.

### Role of inhibition in shaping orchestrated cortical network activity

The high stimulus-related information content found in INH cells at both tested stimulus modalities (location and frequency) suggests that barrel cortex interneurons may be sharper tuned than principal neurons with respect to the different stimuli employed. This conclusion is only apparently in contrast with previous studies, which reported (1) that putative fast-spiking interneurons present larger receptive fields [31] and (2) that inhibition is either similarly tuned as excitation, broader tuned, or not tuned at all [9,31,34,35]. In the present study INH neurons elicited a higher number of spikes than EXC neurons in response to both PW and NW stimulation, thus presenting larger receptive fields [12]–a result not necessarily implying lower stimulus discriminability. Thus, a neuron with a large receptive field can still distinguish between several different stimuli if each stimulus is able to elicit a different and unique activity pattern (a hypothesis strongly supported by our data). The sharp tuning of interneurons has probably been underestimated so far, since none of the previous studies in the auditory [34], visual [9,35] or barrel cortex [31] considered the temporal precision of the responses when computing tuning curves. Therefore, we conclude that the encoding capacity of INH neurons is higher when their high temporal precision is considered (Fig 3), a property consistent with the key role played by INH neurons in synchronizing network activity with millisecond precision [36,37].

Stimulus location information was particularly high in L4 INH neurons, which are the first cortical neurons to receive and process sensory inputs [33,38,39]. The stimulus-related information transferred through feedforward inhibition by this neuronal population can have a strong influence on the firing probability and spike timing of subsequently activated neurons [8,40], a notion supported by the fact that the same afferent fibers make stronger excitatory connections onto INH than EXC cells [33,36]. Here we propose that such a well-defined and temporal precise control over neuronal activity requires a very high level of stimulus-related information.

### Less information redundancy in ensembles of interneurons

Another interesting feature of INH cell ensembles was their lower amount of redundancy as compared to that carried by comparable ensembles containing the best EXC cells. Our results demonstrate that the low redundancy found in INH neuron ensembles was due to the higher variability in their mean stimulus responses, quantified as lower values of the signal similarity term (Table 1). However, the contribution of stimulus-independent noise correlations was very low and not different between INH and EXC cells, thus not playing a major role for stimulus encoding in general or the amount of redundancy in particular [5]. This outcome is consistent with the low level of noise correlations computed between pairs of cells (Fig 6B3), a property which has been previously documented in cortical neurons [41,42]. Our results are also in agreement with those reporting a beneficial effect of low noise correlations on trial-by-trial decoding performance, and thus on stimulus encoding [21,29,30]. It has been suggested that an active decorrelation mechanism is implemented in the cortex in order to reach such low noise correlations [43]. Accordingly, basal forebrain activation has been shown to cause decorrelation between neurons and improve the trial-to-trial response reliability, an effect which is mediated by neuromodulators [44]. Further, several studies have implicated inhibition as a possible decorrelation mechanism [11,45,46].

The distinct connectivity patterns of INH and EXC neurons most likely contribute to the different redundancy levels at which they operate. Cortical INH interneurons are known to have a high dense local connectivity [47]. In contrast, EXC cells in the barrel cortex have more sparse local connectivity, but they can distribute information to remote cortical and subcortical brain areas through long-range projections [48]. While the connectivity patterns of INH neurons favor a precise control of neuronal activity within local microcircuits, EXC neurons convey sensory information to different brain regions for further signal integration and processing. Therefore, having more EXC neurons containing a redundant "message" might be crucial to guarantee the efficient delivery of sensory information to several distinct brain areas [5]. Another reason why redundancy in EXC neurons might be advantageous is that, in the cortex, single excitatory synaptic inputs are very weak [49]. As a consequence, a relatively large number of presynaptic EXC neurons need to fire synchronously (or in a narrow time window) to depolarize the postsynaptic cell to the spike threshold [50].

The information values computed in this study were related to a restricted stimulus set, i.e. containing ≤6 stimulus classes per set. Since the maximum theoretical values of information were therefore rather limited (see Methods), considerable levels of redundancy were reached when increasing the number of neurons in the population (Fig 5C1). Thus, performing the same computations using larger stimulus sets might have an effect on the results, therefore affecting the aforementioned conclusions. However, comparable differences in redundancy between INH and best EXC neurons were obtained when the stimulus set contained 2–3 stimulus classes (stimulus location, Fig 6A), and when it contained 6 classes (stimulus frequency, Fig 8A). Future experiments involving larger sets of more natural stimuli should shed further light into this important issue.

### Effect of anesthesia

All the results described in the present study were obtained from neural recordings performed in animals under anesthesia (see Methods). The major reasons for performing our experiments in anesthetized animals were the following. Firstly, we aimed to perform long experimental sessions, in order to record large numbers of trials in different conditions, including low frequency stimulation (thereby addressing the sampling bias issue, see Methods). However, this would not have been possible in awake animals, since in this case the experimental sessions are usually limited to less than 1 h–i.e. the time that the animals are willing to retrieve water reward [51]. Secondly, we wanted to maintain the conditions for whisker stimulation as invariable and stable as possible, so that an identical sensory input was generated in each trial. Thus, we restricted the sources of external variability to a minimum, to ensure that the variability present in the neuronal responses was only of neuronal origin–i.e. intrinsic to the nervous system [1,27], and not produced by the movements of the animals themselves. Thirdly, we aimed to restrain the variability of spontaneous brain states to a minimum. In this regard, cognitive factors (including working memory and attention) have a significant effect on cortical dynamics [52,53]. For instance, the encoding of sensory inputs has been shown to be state-dependent and apparent only in anesthetized and active awake animals, but not during “quite wakefulness” [54]. Thus, we applied anesthesia in order to avoid undesirable effects produced by uncontrolled cognitive variables on the neuronal responses.

Although our experiments were performed under anesthesia following the aforementioned reasons, we expect that the effects described in our study are also present during wakefulness. The level of inhibition in granular and supragranular layers has been shown to be higher during wakefulness and lighter states of anesthesia than during deep anesthesia, thus producing lower firing rates in pyramidal cells [40,55,56]. This might be a mechanism to optimize the extraction of behaviorally relevant signals by L2/3 neurons from the otherwise noisy cortical activity [55,56]. We argue that this higher level of inhibition could further enhance the level of information encoded by INH neurons with respect to that encoded by EXC neurons. Moreover, comparable numbers of evoked action potentials have been observed in previous studies performed in the barrel cortex of awake rodents [57,58]. Concordantly, the neural latencies in the principal column were not affected by the level of anesthesia [59]; in addition, the differences in the number of action potentials evoked by PW vs. NW deflections (which determines the stimulus selectivity of spike counts) were not considerably affected either [59]. All these data suggest that our results are not necessarily different to those that could have been obtained in awake animals. Future experiments performed in different behavioral and cognitive states should systematically address this issue in more detail.

### Pathways for processing spatially and temporally encoded information

Our data are compatible with the concept that different thalamocortical pathways perform diverse computations in parallel, although terminating in the same cortical column. It has been previously proposed that while the lemniscal system (to which L4 belongs) processes spatially encoded information (i.e. stimulus location), the paralemniscal system (L5A) processes temporally encoded information (i.e. stimulus frequency) [25,60]. Our results might extend this notion by showing that while L4 INH neurons carry the highest amount of stimulus location information, INH neurons in granular and infragranular layers do so for stimulus frequency information, thus indicating that the information processed in the network was dominated by INH neurons at both stimulus modalities.

In line with this, one intriguing outcome is the fact that EXC neurons do not follow a similar distribution of information content across layers as INH cells (Fig 3B & 3F). Of particular interest is the low amount of stimulus location related information carried by L4 EXC cells (Fig 3B), a result which is however consistent with their low number of spikes elicited in response to both PW and NW stimulation [12,61]. Another intricate result was the lower amount of stimulus location information carried by barrel cortex neurons at behaviorally relevant frequencies (4–10 Hz) [62,63], both at the individual neuron (Fig 3C and 3D) and ensemble (Fig 7B) levels. Three explanations (not mutually exclusive) could account for these unexpected findings. First, it is most likely that active whisking regulates the ongoing activity in the barrel cortex of behaving animals, so that the trial-to-trial evoked responses are optimized, a mechanism which would not be present during passive whisker stimulation [55,57]. Second, it is possible that secondary sensory or motor areas perform an integration of the sensory-evoked information over a time period including several stimulation trials, thus being specifically enhanced at higher stimulation frequencies [64]. Third, at physiological whisking frequencies the cortical information processing might rely on multi-whisker inputs, rather than on single-whisker stimulation. Future experiments should systematically address these issues in order to test their relevance and functional implications.

Further, an alternative strategy could be proposed for the representation of stimulus information at higher frequencies, rather based on the time interval between consecutive population responses. The stimulus frequency might be decoded by measuring the time between two population events, i.e. by dynamically computing the inter-event-intervals between stimuli. We consider this possibility unsuitable for the following reasons. Firstly, previous studies have shown that a time window of ~50 ms is optimal for decoding both the location and frequency of whisker stimuli [3,20,21,25,30,65]. Importantly, this time window coincides with the first volley of activity elicited within the barrel cortex from the thalamo-cortical inputs, which does not last longer than ~50 ms from stimulus onset in any layer or neuronal type [26,61,66]. Secondly, population responses considerably change in their amplitude and temporal precision for the different stimulus frequencies (Figs 7 and S4) [23–26]. As a consequence, discriminating population events from spontaneous activity becomes increasingly difficult at higher stimulation frequencies, thus making very unlikely a strategy in which the time between population events is quantified. Thirdly, the neuronal mechanisms necessary to perform such time-based computations would probably involve working memory, and further information processing performed in cortical or subcortical areas receiving projections from the barrel cortex [60]. In order to avoid such assumptions, we focused on the stimulus information encoded in each trial by barrel cortex neurons, i.e. limiting ourselves to the first volley of sensory-evoked activity. Our results are thus in agreement with stimulus encoding based in firing rate [25,26], and also with current theories characterizing the feedforward propagation of synchronized neural activity within the cortex [67].

## Materials and Methods

### Ethics statement

All procedures were approved by the local ethics committee (#23177-07/G10-1-010), and followed the European and German national regulations (European Communities Council Directive, 86/609/ECC).

### Surgery, recording and stimulation protocols

A detailed description of the experimental protocols can be found in a previous study reporting different but complementary analyses performed on the same experimental data [12]. Male Wistar rats (postnatal day 28–42, 80–200 g) were anesthetized using urethane (1.5 g/kg) and head-fixed into a modified stereotaxic device. A 3x3 mm^2^ craniotomy was performed over the barrel cortex of the left hemisphere. Body temperature was held at ~37°C. The depth of anesthesia was maintained at stage III/3–4 [68].

The identification of the barrel-related columns of interest was performed by voltage sensitive dye (VSD) imaging using the VSD RH1691 (Optical Imaging, Rehovot, Israel). VSD signals were evoked by single whisker deflections and collected using the same procedures as previously described [12], determining the insertion position of the 16 or 128 channel ‘silicon probes’ (NeuroNexus Technologies, Ann Arbor, MI, USA). The one-shank 16 channel probe had a separation of 100 μm between recording sites. The 128 channel probe contained 8 shanks separated by 200 μm, presenting a vertical spacing of 75 μm between the recording sites (Fig 1B). For histological verification of tracks, the probes were labeled with DiI (Molecular Probes, Eugene, OR, USA) before insertion.

Individual whiskers were briefly deflected at 5 mm from their base by inserting each of them into a capillary tube glued to a piezoelectric bimorph actuator (Physik Instrumente, Karlsruhe, Germany) which was controlled by a voltage pulse generator (Master-8, A.M.P.I., Jerusalem, Israel). The voltage pulse was an up-down square step function of 2 ms duration, producing a capillary movement of amplitude 150 μm with a rise time of 1.5 ms (measured at the capillary ending). The following stimulation protocol was repeated for each of the selected (2 to 3) target whiskers. First a block of stimuli was applied at low-frequency, typically containing 200 trials with an inter-trial-interval of 5 to 30 s (thus corresponding to stimulation frequencies between 0.03 and 0.2 Hz). Afterwards blocks of stimuli were applied at higher frequencies (1, 2, 4, 7 and 10 Hz), each of them containing 100 trials. Individual blocks of trials were separated by periods of at least 10 s. After these sets of stimulations spontaneous activity was recorded for 500 to 2000 s. All data were continuously digitized at 20 kHz and stored for offline analysis.

After the experiment, animals were deeply anesthetized with ketamine (120 mg/kg) and xylazine (5 mg/kg) and perfused through the aorta with 4% paraformaldehyde. In order to visualize the barrel field, 80 μm thick histological sections were prepared and processed for cytochrome oxidase (CO) histochemistry. To verify the columnar location of the recorded neurons, the individual probe shanks (previously labeled with DiI) were identified relative to the position of the barrels within the histological sections.

### Data analysis

Unless otherwise stated, all analyses were performed offline using custom programs written in Matlab (Mathworks, Natick, MA, USA) and C. Raw data (digitized at 20 kHz) were accessed using functions included in the FIND Matlab toolbox [69]. For spike detection and sorting (see below) signals were used at full sampling rate. For local field potential (LFP) analyses they were downsampled to 1 kHz.

Multi-channel based spike detection and sorting was performed as described previously [12,70]. In brief, the continuously recorded raw signals were high-pass filtered (0.8–5 kHz). Non-overlapping groups of 2–4 contiguous channels (potentially recording from the same neurons) were selected as ‘virtual tetrodes’. Spike detection was performed in each group of channels independently using amplitude-thresholding. Extracted spikes contained the sampled amplitude values from all channels in the group in the time range from -0.5 to +0.5 ms relative to the waveform negative peak. From the spike waveforms, we then computed feature vectors containing three values for each channel (the negative peak amplitude plus the two first principal components derived from the waveforms). The (n*3)-dimensional vectors (where n represents the number of channels within a group) were sorted using KlustaKwik (http://klustakwik.sourceforge.net) and Klusters (http://klusters.sourceforge.net) [71,72]. Several criteria were established in order to ensure the isolation quality of the sorted neurons, accounting for (1) a clear refractory period present in the activity of the isolated units, (2) a stable spontaneous firing rate during the whole duration of the recordings, and (3) a valid “isolation distance” reached during the spike sorting procedure [12]. Cells were subsequently classified as putative inhibitory (INH) and excitatory (EXC) neurons based on their mean spike waveform [16,17].

The cortical depth of the individual channels was assessed from the stereotaxically estimated depth of the probe tip and the current source density (CSD) maps computed from the LFPs. We used the early CSD sinks present at the thalamo-recipient L4 and L5B/6 around 6 to 10 ms after sensory stimulation in order to assign the individual channels to specific cortical layers [12]. In case the action potentials of a spike-sorted neuron were detected in more than one channel, the somatic location of the spike-sorted neuron was assigned to the recording site containing the mean waveform with maximum negative peak amplitude.

To display the overall changes in the activity of individual neurons, peri-stimulus time histograms (PSTHs) were computed using a time resolution of 2.5 ms (Fig 2). Spontaneous activity was estimated for every neuron by computing their mean firing rate (FR) during the whole recording time, excluding the 1 s time window after each whisker stimulus. Evoked activity was firstly analyzed by computing both the mean spike counts and the median first-spike latencies within the time window from 0 to 50 ms after each stimulus (S4 Fig).

### Mutual information

Information theory was used to quantify the amount of information conveyed by either individual neurons or by neuronal ensembles related to the stimulus properties under consideration (location and frequency, see below) [14]. Mutual information (*I*_*m*_) was computed as:

$$I_{m}(S;R)=H(R)-H(R|S)=\sum_{s\in S}P(s)\sum_{r\in R}P(r|s){\text{log}}_{2}\frac{P(r|s)}{P(r)}$$

where *H*(*R*) represents the overall variability of the responses (i.e. response entropy), and *H*(*R* | *S*) represents the trial-to-trial variability in the responses to a given stimulus (i.e. noise entropy). *P*(*r*) is the probability of observing the response *r*, *P*(*r* | *s*) the probability of observing the response *r* when the stimulus *s* is presented, and *P*(*s*) the probability of presentation of the stimulus *s*, which is computed as:

$$P(s)=\frac{N_{tr}(s)}{N_{tr}^{tot}}$$

where *N*_*tr*_ (*s*) is the number of trials available for the stimulus *s*, and $N_{tr}^{tot}$ is the total number of trials.

Two stimulus modalities were independently used as inputs in our equations: stimulus location (i.e. specific whisker deflected) and stimulus frequency (i.e. frequency of the deflections given to a single whisker). In the case of stimulus location, the number of elements in *S* differed for individual neurons or ensembles. For individual cells, responses evoked by stimulating the principal whisker (PW) were compared with responses evoked by stimulating any of the adjacent neighboring whiskers (NWs). This in agreement with the notion that the best encoded stimulus location in barrel cortex neurons is their PW [3]. As a consequence, the numbers of elements in *S* was always 2, and the *I*_*m*_ could reach a maximum value of 0.98±0.1 bits (Figs 1, 2, 3A–3D, S2 and S3)–note that the number of trials was not always the same for all stimulus conditions, and therefore the actual values were lower than the maximum theoretical ones (log_2_ (2) = 1 bit). For neuronal ensembles, the numbers of elements in *S* ranged between 2 and 3 (i.e. depending on the total number of whiskers stimulated), and therefore the maximum value for the ensemble mutual information (*EI*_*m*_) was 1.18±0.1 bits (Figs 5, 6, S7 and S8A–S8C). To quantify information related to the stimulus frequency, we compared the responses evoked by stimulating a given whisker at different frequencies (<1, 1, 2, 4, 7, 10 Hz). In this case the number of elements in *S* was 6 and *I*_*m*_ yielded a maximum value of 2.52±0.01 bits (Figs 3E, 3F, 8A and S8D).

Two different measures were used to represent the whisker-evoked responses: (a) spike counts and (b) spike patterns. Spike counts were counted for increasing window lengths (S2A Fig); given a specific window length (50 ms), spike patterns were generated by subdividing the 50 ms time window into smaller time bins (S2B Fig). Intuitively, spike patterns generated using shorter time bins achieved a higher temporal resolution, which led to a higher discriminability and therefore increased values of mutual information (Fig 1D). In this regard, for individual neurons the response to a stimulus was represented either by one value (spike count), or by a spike vector with a number of elements *N*_*bins*_ = *T*_*window*_ / Δ*t*, where *T*_*window*_ is the duration of the response window and Δ*t* the bin size. On the other hand, each neuronal ensemble response was represented by a multidimensional array of *N*_*neurons*_ ⋅ *N*_*bins*_ dimensions (Fig 4B).

Mutual information was computed for variable-sized neuronal ensembles in each experiment. Specifically, we created neuronal ensembles of increasing size, starting with one single neuron and adding one neuron at a time until the whole neuronal population was included. Two different orderings of neural selection were considered, (1) ascending ordering, adding in each step the least informative neuron to the ensemble (Fig 5B1), and (2) descending order, adding in each step the most informative neuron (Fig 5B2). This procedure was based on the level of information conveyed by the neurons individually, which was previously computed (Fig 3B & 3F).

One of the major issues within the information theory framework is the presence of a systematic error (called bias), as a consequence of the limited number of stimulation trials that is possible to record during an experimental session [73]. Our recorded datasets contained typically 200 trials (repetitions) when the whiskers were stimulated at low frequency (<1 Hz), and 100 trials at higher frequencies (from 1 to 10 Hz)–note that at stimulations frequencies ≥4 Hz the first 20 trials were discarded due to the presence of neural adaptation (see Results). In general, there is bias when the number of elements representing the responses (i.e. within the response vectors or matrices) is similar or higher than the number of individual trials. For instance, for neuronal ensembles of 10 neurons and 10 time bins (50 ms long windows / 5 ms resolution) spiking a maximum of 1 spike per time bin, there are (at most) 1024 different possible patterns of each neuron, and therefore 10^1024^ different responses. This large response space cannot be entirely sampled by the recorded neurons within the given number of trials, and as a consequence the computed values of mutual information are estimated to be higher than their actual values. However, note that the dimensionality reached by the response space of the spike patterns is most likely not as high, since the majority of cells elicit <1 spike per stimulus (S4 Fig). Therefore, a spike pattern containing 1 spike in each of its time bins is very unlikely to occur and, as a consequence, the real number of possible spike patterns of each neuron is actually <<1024.

In order to address this issue, we explored the performance of several bias correction methods previously published and implemented in the STAToolkit Matlab toolbox [74]. We explored the effect of increasing the number of elements within the response vectors (and matrices) on the corrected mutual information values (computed using six different bias correction methods) (S7 Fig). The QE [75] and jackknife [76] methods delivered the most accurate results, followed by the Wolpert-Wolf [77]. From them, the QE method was selected because it has been widely employed and well-documented, delivering a performance similar to that of the Panzeri-Treves method [78,79]. Other methods, such as the best upper bound [80], the Chao-Shen [81] and the Nemenman-Shafee-Bialek [82] methods yielded inaccurate (i.e. too high or erroneous) information values and were discarded from further analysis.

Further, we characterized the decay in mutual information values when progressively larger numbers of trials were used for their computation. In agreement with previous studies [73,79], the amount of computed information decayed asymptotically with increasing number of trials (S8 Fig). Similar results were obtained when networks of INH or best EXC neurons were considered. As expected, the number of trials necessary to approach asymptotic values was lower as the number of elements in the response vectors (or matrices) decreased–i.e. when smaller numbers of neurons in the ensemble were selected, or when spike counts (instead of spike patterns) were used for quantification. This analysis confirmed that the number of trials used was sufficient to correctly estimate the true values of mutual information in small neural networks (i.e. containing ≤10 neurons), and therefore to obtain conclusions about their respective encoding strategies (see below)–note that the majority of the networks considered in detail in this study contained ≤10 neurons (Figs 6B2 and 8A2).

Further, the information values computed here using spike patterns (termed “full mutual information” in previous publications) might be regarded as an upper bound for the true information encoded by the neuronal network [28]. Nevertheless, it has been shown that (1) under certain circumstances the full mutual information can be accurately corrected for bias, and that (2) putative physiological decoders could implement heuristic mechanisms to extract the full amount of information provided by such representation [28]. A lower bound for the true information conveyed by the network was estimated by quantifying the LDA performance using the transmitted information (i.e. the mutual information of the confusion matrix between real and decoded stimulus class). In all cases the stimulus related information was higher when computed using the direct (debiased) method than when computed using the transmitted information (see Results).

The contribution of signal and noise correlations on the total mutual information was quantified as described in Pola et al. [14]:

$$I_{m}(S;R)=I_{lin}+syn=I_{lin}+I_{sig-sim}+I_{cor}=I_{lin}+I_{sig-sim}+I_{cor-ind}+I_{cor-dep}$$

The linear term *I*_*lin*_ is the sum of the information provided by each individual cell, which is computed as:

$$I_{lin}=\sum_{c}\;\sum_{s\in S}P(s)\sum_{r_{c}\in R_{c}}P(r_{c}|s){\text{log}}_{2}\frac{P(r_{c}|s)}{P(r_{c})}$$

The remainder between *I*_*m*_ (*S*; *R*) and *I*_*lin*_ is called synergy (*syn*). When this term has a positive value, it indicates the presence of synergistic interactions between the neurons, thus making the information conveyed by the neuronal ensemble as a whole higher than the sum of the information provided by the neurons individually. In contrast, negative values indicate that individual neurons carry redundant information.

The differences in information redundancy can be derived from distinct stimulus encoding strategies used by the neuronal populations. Generally, a lower redundancy is obtained (1) when neurons present more variable mean stimulus responses (quantified in the level of signal correlations), and/or (2) when neurons represent stimuli using cooperative mechanisms (manifested in the structure and level of noise correlations). We evaluated these possibilities by applying the full information-breakdown methodology. Thus, the synergy term was further divided into the signal-similarity term (*I*_*sig*−*sim*_) and the noise correlation term (*I*_*cor*_). Specifically, the signal-similarity term (*I*_*sig*−*sim*_) quantifies the impact of signal-correlations between neurons on the ensemble-based mutual information, presenting always negative values (i.e. being detrimental for the information conveyed by the ensemble). The noise correlations term (*I*_*cor*_) quantifies whether the presence of noise correlations increases or decreases the information available in the population responses, compared to the case where such correlations are absent, therefore being either beneficial (positive) or detrimental (negative). Please note that in the present study we only tested the effect of cross-cell noise correlations on the ensemble-based information, thereby disregarding the presence of within-cell correlations [5].

We further segregated the partial contribution of the stimulus-independent correlational term *I*_*cor*−*ind*_ (reflecting the contribution of stimulus-independent correlations) and the stimulus-dependent correlational term *I*_*cor*−*dep*_. The specific formulae employed for the computation of the terms *I*_*sig*−*sim*_, *I*_*cor*−*ind*_ and *I*_*cor*−*dep*_ are described in detail in previous publications [14]. All term values reported in the present study were computed using the information breakdown Matlab toolbox (ibTB) [83].

### Linear discriminant analysis classifier

Linear discriminant analysis (LDA) is a standard supervised classification technique, which has been shown to provide the best performance with the lowest computational cost in experimental conditions similar to ours [65]. In brief, LDA generates a set of linear discrimination functions from a set of multiple independent variables that best separates the groups of classes–assuming that the multivariate data are normally distributed, with equal covariance matrix for each class [84].

In our data, the number of classes (termed |*S*| following the notation used for mutual information) derives from the stimulus property being classified, i.e. stimulus location or frequency. The independent variables derive from the recorded ensemble responses (termed *r* above), being represented by (a) spike counts or (b) spike patterns (see above). Because LDA requires a preprocessing step of dimension reduction, we applied principal component analysis (PCA) on the input data [13]. Thus, each neuronal ensemble response was represented by the ten first principal components derived from the whole response array *r* of *N*_*neurons*_ ⋅ *N*_*bins*_ dimensions.

Each of the discriminant functions is defined by a set of weights (*w*) associated with each of the independent variables (*x*):

$$y_{k}(x)=w_{1}x_{1}+w_{2}x_{2}+\cdots +w_{10}x_{10}$$

where *k* = 1⋯|*S*|−1, *w* are the discriminant weighs, and *x* are the principal components derived from the response matrices. These discriminant functions were computed using a subset of the trials (training trails). Another subset of the trials (testing trials) was used to evaluate how well the stimulus properties could be predicted by the LDA classifiers on a single-trial basis. In this regard, four-way cross-validation was used to evaluate the decoding performance. For each classifier, the trials set was divided into four equally-sized, mutually-exclusive subsets, and the discrimination functions were generated using three of the subsets as the training set (i.e. 75% of the trials). The remaining trials (25%) were used as the test set. In this way, each data set was used three times in training and once in testing. The resulting performance was then averaged from the outputs of all the test data.

To test the dependence of decoding performance on the size of the considered population we applied the same incremental procedure of neural selection as the one described for mutual information (see above) (Fig 5A). In this regard, the neural selection process was again based on the level of information conveyed by the neurons individually.

We further tested the role of correlated activity among neural spike counts or patterns (i.e. stimulus-independent cross-cell noise-correlations) in the performance of our LDA classifiers. To this end, trial-shuffling surrogate data were generated by randomly replacing the raw spike trains of each neuron with those from another trial. Afterwards the same procedure for training and testing (four-way cross validation) was used as described above.

### Statistics

To test the hypothesis that two samples represent similar distributions, first each distribution of data was tested for normality (D’Agostino-Pearson test) within each group. If data samples were normally distributed and had a size ≥20, parametric tests (independent or paired t-test) were applied. Otherwise nonparametric tests (Mann-Whitney U or paired Wilcoxon signed-rank test) were performed (5% significance level). To test for significant differences between three or more neuronal groups, non-parametric permutation-based ANOVA tests were used, with posthoc false discovery rate (FDR) correction for multiple comparisons [85,86]. Unless otherwise stated, values throughout this report are given as mean±SEM.

## Supporting Information

S1 Dataset Spike and stimulus timing data for all animals and experimental conditions. (ZIP)

S1 Fig Classification of putative inhibitory and excitatory neurons based on the mean spike waveforms. (PDF)

S2 Fig Effect of the window length selected for spike counting and bin size of spike patterns on stimulus location information. (PDF)

S3 Fig Time course of stimulus location information carried by the spikes counted in increasingly longer time windows at 1 Hz, 4 Hz and 10 Hz stimulation frequencies. (PDF)

S4 Fig Mechanisms underlying stimulus discriminability in inhibitory and excitatory neurons across layers. (PDF)

S5 Fig Decoding performance achieved by the linear discriminant analysis classifiers. (PDF)

S6 Fig Effect of the window length and bin size used to generate spike counts and patterns on the resulting stimulus frequency decoding performance. (PDF)

S7 Fig Effect of the number of elements representing neural responses on bias corrected mutual information values. (PDF)

S8 Fig Effect of the number of trials used to compute bias corrected mutual information values. (PDF)
