# Supplementary material for: High Stimulus-Related Information in Barrel Cortex Inhibitory Interneurons
Source: PLoS Comput Biol. 2015 Jun 22;11(6):e1004121. doi: 10.1371/journal.pcbi.1004121 (PMC4476555; doi:10.1371/journal.pcbi.1004121)
Supplement: S1 Fig — (PDF) [file pcbi.1004121.s002.pdf]

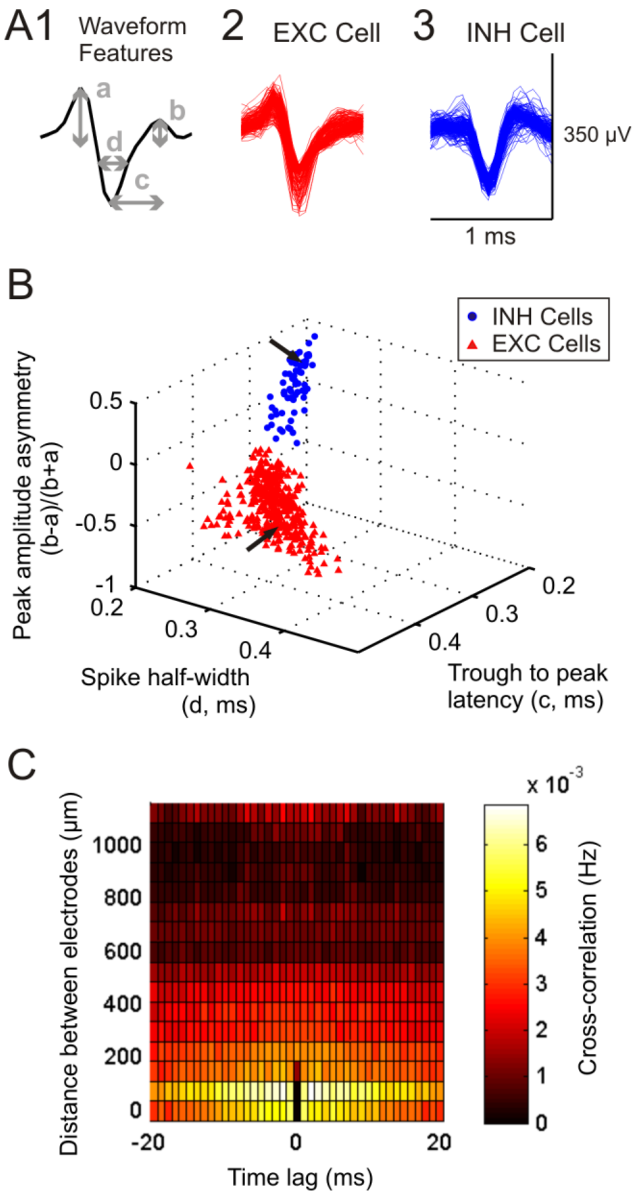

Figure S1. Classification of putative inhibitory (INH) and excitatory (EXC) neurons based on the mean spike waveforms. For each neuron three parameters were calculated, which have been shown to reliably separate between several identified neuronal populations [15–17]: (1) the trough to right (late) peak latency (related to the repolarization of the intracellular action potential), (2) the spike half width, and (3) the peak amplitude asymmetry index (possibly reflecting differences in the rate of fall of spike repolarization). **(A1)** Schematic drawing illustrating the three features extracted from the spike waveforms to perform neural classification: a and b, left (early) and right (late) baseline-to-peak amplitudes; c, trough to right peak latency; d, spike half width. The first two features were used to calculate the peak amplitude asymmetry index [(b-a)/(b+a)]. **(A2&3)** Waveforms extracted from the typical EXC and INH neurons marked in B with black arrows. **(B)** Clustering of the two neuronal groups (putative EXC and INH neurons) by the k-means algorithm using the three parameters described in A1. The resulting automatic separation was

similar to the one previously published [12]. **(C)** Mean cross-correlations computed between single units located at different inter-electrode distances along the electrode shanks, including periods containing both sensory-evoked and spontaneous activity. Coincidence rate values were averaged from all neurons sharing a similar inter-electrode distance on the same shank (i.e. within the vertical direction). In order to establish standard inter-electrode distances, we only included in this analysis the experiments in which the 8x16 electrode type was employed (n=9 animals, 410 neurons). The highest correlation values were found in neurons recorded in neighboring channels (~75 µm apart) at a time lag of ~4 ms. Note that the cross-correlograms were symmetric because they were averaged by comparing the neurons in both directions (i.e. neuron A vs. B and B vs. A). The absence of high correlation values at time lag 0 demonstrate the lack of cross-contamination due to errors in spike sorting, i.e. the assignment of the same event to two different units.

Note the low correlation values present at time lag 0 within the same electrode or closely neighboring electrodes. This feature is due to a well-known limitation of the multi-electrode spike sorting technique, which (by performing the sorting in subsets of 2–4 neighboring channels) is able to resolve the occurrence of distant overlapping spikes, but not local ones [12,70,71]. Thus, if two closely spaced neurons are active synchronously (i.e. within the time range of ±0.5 ms), a subset of closely neighboring electrodes (which have overlapping 'sampling volumes') will register the sum of the spikes of the two neurons, an occurrence which is too sporadic to be adequately classified.

Across all cortical layers, INH neurons presented higher spontaneous activity than their EXC counterparts. The spontaneous firing rate (FR) was distributed among the established neuronal groups as follows: L2/3 INH, 0.8±0.4 spks/s; L2/3 EXC, 0.3±0.05 spks/s; L4 INH, 1.2±0.3 spks/s; L4 EXC, 0.5±0.07 spks/s; L5A INH, 2±0.7 spks/s; L5A EXC, 1.6±0.1 spks/s; L5B/6 INH, 1.9±0.3 spks/s; L5B/6 EXC, 1.1±0.1 spks/s. The results of the permutation test indicated a significant effect of the layer ($p<0.01$) and the type of neuron (INH/EXC) ($p<0.05$) on the spontaneous FR, but not of their interaction ($p=0.99$). Thus, spontaneous FR was consistently higher in INH than EXC neurons across all layers, being highest in L5A and lowest in L2/3 neurons.
