# Supplementary material for: High Stimulus-Related Information in Barrel Cortex Inhibitory Interneurons
Source: PLoS Comput Biol. 2015 Jun 22;11(6):e1004121. doi: 10.1371/journal.pcbi.1004121 (PMC4476555; doi:10.1371/journal.pcbi.1004121)
Supplement: S2 Fig — (PDF) [file pcbi.1004121.s003.pdf]

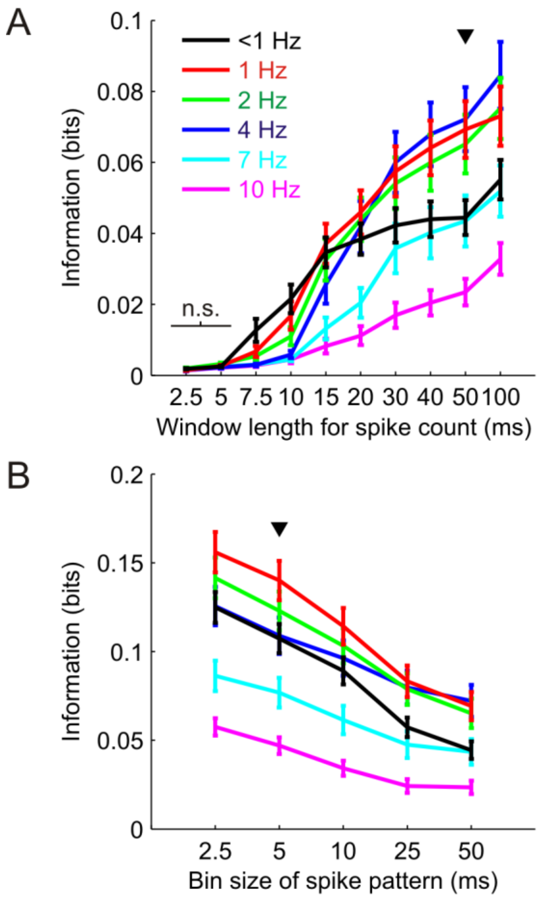

Figure S2. Effect of the window length for spike counting and bin size of spike patterns on stimulus location information. (A) Effect of the total window length used to generate spike counts on the resulting information. Data represent mean±SEM of the stimulus location information at a specific stimulation frequency averaged across all available cells (see below for further details). The values across stimulation frequencies were not significantly different when the window length was 5 ms or lower. Black downward triangle indicates the window length selected for further analysis (50 ms). **(B)** Given a window length of 50 ms, effect of the bin size used to generate spike patterns on the information at the single cell level. In this case, differences across stimulation frequencies were significant at all tested bin sizes (see below).

In panel A, we compared for each cell the responses when its PW was stimulated versus when any of its adjacent NWs was stimulated, thereby determining its stimulus location information. In this computation the values from all recorded neurons were pooled together, regardless of the neuronal type (INH, EXC) or layer (low frequency stimulation, n=263 neurons from 10 animals; higher frequencies, n=187 neurons from 7 animals). As expected, within the first 5 ms after whisker-stimulus no information was present in the spike counts of barrel cortex neurons. The subsequent gradual increase in window length caused a continuous information growth, reaching a value of 0.044±0.005 bits within 50 ms after stimulus for low frequency stimulation – note that the differences across frequencies were highly significant

from a time window of 7.5 ms onwards (permutation test, $p<0.001$). The growth rate of the information however decreased after 50 ms post-stimulus, presenting 0.055±0.006 bits at 100 ms post-stimulus (note that the values presented at the x-axis are not linearly scaled). The values obtained for the remaining stimulation frequencies presented similar temporal dynamics, being at 50 ms post-stimulus highest for the 4 Hz frequency (0.072±0.009 bits) and lowest for the 10 Hz frequency (0.023±0.004 bits). These results, together with those obtained in the analyses based on LDA classifiers (Figure S5), suggested to select 50 ms as an adequate window length for optimal stimulus location discrimination – a choice in agreement with previous publications [3,21].

Once the window length for characterizing neuronal responses was set to 50 ms, we further tested the influence of the spike patterns bin size on mutual information (panel B). As expected, and in agreement with previous reports [3], increasing the temporal resolution of the generated spike patterns by using smaller bin sizes, led to a gradual enhancement in the stimulus discrimination capacity (here quantified as mutual information). The differences across frequencies were highly significant at all bin sizes (permutation test, $p<0.001$). Specifically, mutual information increased when the frequency of whisker stimulation was decreased from 10 to 7, 4, 2 and 1 Hz. Nonetheless and surprisingly, when the stimulation frequency was below 1 Hz we observed a decrease rather than a further increase in mutual information. This finding suggests that 1–2 Hz stimulation frequencies are optimal for barrel cortex neurons in order to encode the location of passive whisker stimuli. At 5 ms bin size the mutual information reached a value of 0.11±0.008 bits for low frequency stimulation, being highest for 1 Hz stimulation (0.14±0.011 bits) and lowest for 10 Hz (0.047±0.005 bits). We selected this bin size for further analyses, which is consistent with our results based on LDA classifiers (see below), as well as with those obtained in previous publications [3,11,21].
