# Supplementary material for: High Stimulus-Related Information in Barrel Cortex Inhibitory Interneurons
Source: PLoS Comput Biol. 2015 Jun 22;11(6):e1004121. doi: 10.1371/journal.pcbi.1004121 (PMC4476555; doi:10.1371/journal.pcbi.1004121)
Supplement: S3 Fig — (PDF) [file pcbi.1004121.s004.pdf]

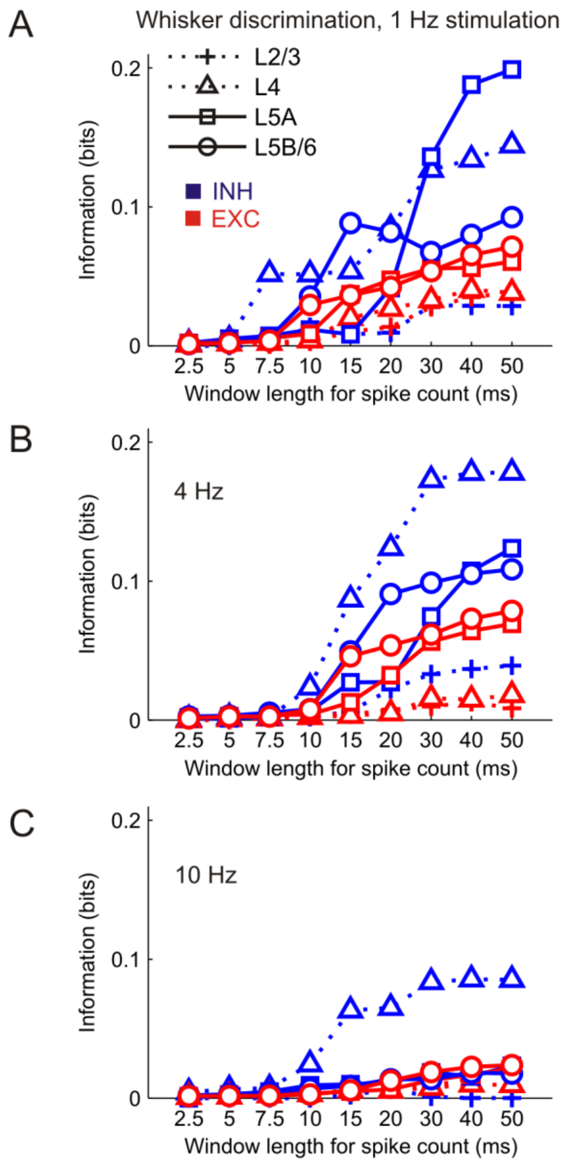

Figure S3. Time course of stimulus location information carried by the spikes counted in increasingly longer time windows at (A) 1 Hz, (B) 4 Hz and (C) 10 Hz stimulation frequencies. In each panel, lines represent the mean values for the different neuronal groups (symbols at the legend in panel A). For each neuron we derived the stimulus location information value by comparing its response distributions to PW vs. NW stimulation (as in Figures 3A–D and S2, see also Figure 1D) (low frequency stimulation, n=263 neurons from 10 animals; higher frequencies, n=187 neurons from 7 animals). Note that at 1 Hz stimulation frequency a linear succession was found in the information peak of the different neuronal populations (panel A); however, at higher stimulation frequencies a rather parallel increase of information was obtained (panel C).
