# Supplementary material for: High Stimulus-Related Information in Barrel Cortex Inhibitory Interneurons
Source: PLoS Comput Biol. 2015 Jun 22;11(6):e1004121. doi: 10.1371/journal.pcbi.1004121 (PMC4476555; doi:10.1371/journal.pcbi.1004121)
Supplement: S4 Fig — (PDF) [file pcbi.1004121.s005.pdf]

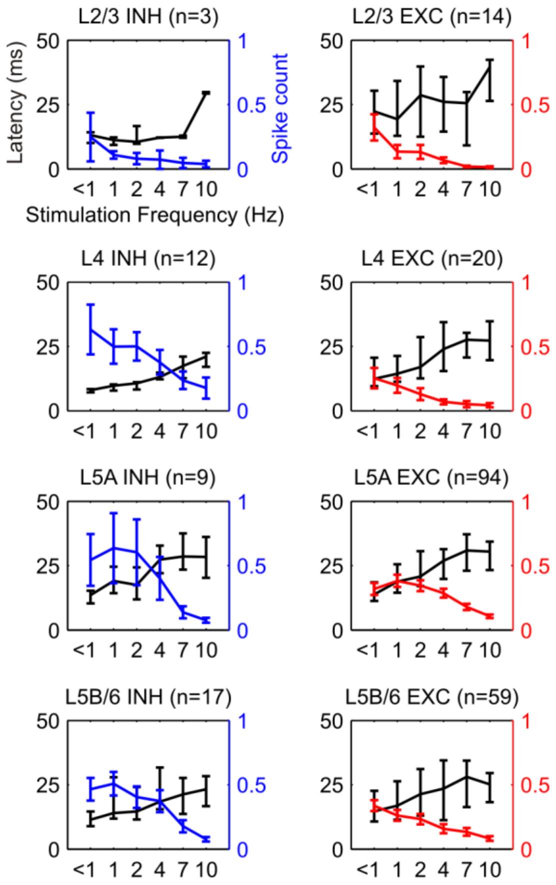

Figure S4. Mechanisms underlying stimulus discriminability in inhibitory (INH) and excitatory (EXC) neurons across layers. For each neuronal group, first-spike latencies and spike counts in a 50 ms time window after PW stimulation are presented at all tested frequencies. Latencies (black lines) are represented by the median and inter-quartile range; spike counts (blue for INH and red for EXC neurons) by mean±SEM. The median and interquartile range are used to represent data related to neural latencies, in order to avoid the bias introduced by these time-based values, which generally present skewed distributions.

In order to investigate the mechanisms responsible for the high stimulus frequency discrimination of L4 and L5A INH neurons, we computed for each neuronal group the median first-spike latencies and the mean evoked spike counts in a 50 ms time window after PW stimulation at all frequencies. In this computation we used only those neurons for which the PW was stimulated at all tested frequencies (< 1, 1, 2, 4, 7 and 10 Hz) (n=228 neurons from 12 animals, similar to Figure 3E–F). In all neuronal groups there was a progressive reduction in spike counts, and a progressive increment of neuronal latencies as the stimulus frequency increased (note that for some neuronal groups this progression started at 2 Hz). For each neuron we further estimated its frequency sensitivity as the SD of the spike counts distributed across stimulus frequencies. Thus, higher values of frequency sensitivity indicate potentially higher values for stimulus frequency information. L4 and L5A INH neurons presented the highest values of frequency sensitivity in their spike counts (0.28±0.06 and 0.29±0.11 spks/stim, respectively). The result of the permutation-based two-way ANOVA

test also indicated that INH neurons presented in general more frequency sensitivity in their spike counts than EXC cells ($p<0.05$).

A similar quantification was applied to the first-spike latencies. In this case, INH and EXC neurons presented rather similar frequency sensitivity in their latencies (permutation test, factor neuronal type, $p=0.12$), being highest in L5A EXC cells (9.08±0.31 ms). Nevertheless, L5A INH cells showed the highest values of all INH groups (9.04±1.18 ms), being higher than in L4 INH cells (5.3±1.16 ms) (not significantly, false discovery rate, $p>0.05$).

These results suggest that L4 and L5A INH neurons are best suited for discriminating between different stimulus frequencies (Figure 3F), since they present the highest frequency selectivity in their spike counts (and therefore the highest information under a rate code scheme). The selectivity based on the first-spike latencies was not a major factor in the resulting frequency information, suggesting that stimulus frequency was encoded using a scheme closer to rate coding.
