# Supplementary material for: High Stimulus-Related Information in Barrel Cortex Inhibitory Interneurons
Source: PLoS Comput Biol. 2015 Jun 22;11(6):e1004121. doi: 10.1371/journal.pcbi.1004121 (PMC4476555; doi:10.1371/journal.pcbi.1004121)
Supplement: S5 Fig — (PDF) [file pcbi.1004121.s006.pdf]

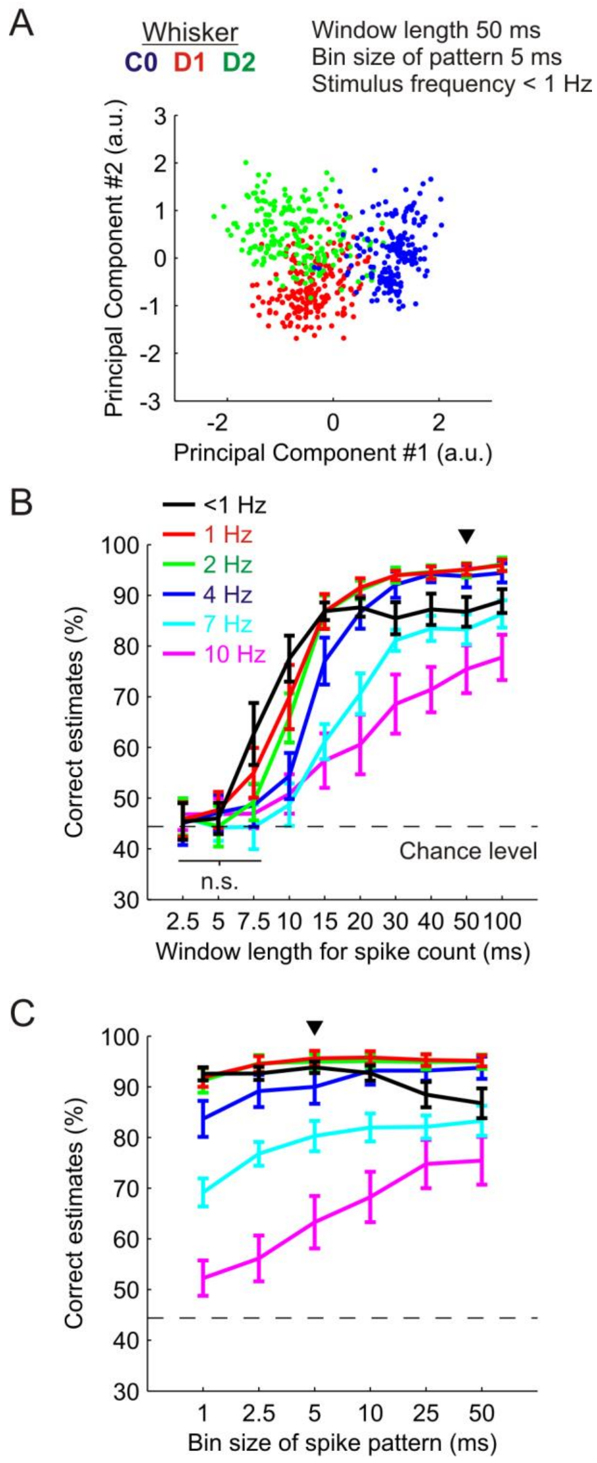

Figure S5. Decoding performance achieved by the linear discriminant analysis (LDA) classifiers. (A) Two first principal components computed with spike pattern arrays from the experiment presented in Figure 4 (n=44 neurons, 50 ms window length, 5 ms time bin). Each single dot represents the neural ensemble response to a single trial of whisker stimulation (200 trials per whisker). Colors represent stimulus location (i.e. the different whiskers stimulated) (see below for further details). **(B)** Effect of the total window length used to generate spike counts on the resulting stimulus location decoding performance. Each line

represents mean±SEM of the stimulus location decoding performance at a specific stimulation frequency computed using all available experiments (n=9 animals for low frequency, n=6 for higher frequencies). The values across stimulation frequencies were not significantly different when the window length was 7.5 ms or lower. Black downward triangle indicates the window length selected for further analysis (50 ms). Dashed horizontal line marks mean chance level of all experiments (44.4%, n=9 animals). **(C)** Given a window length of 50 ms, effect of the bin size used to generate spike patterns on the decoding performance. Differences across stimulation frequencies were significant at all tested bin sizes (see below).

When displaying the two first principal components derived from spike pattern arrays, the ensemble responses formed clusters grouped by the stimulated whisker (n=44 neurons from one animal) (panel A, same illustrative experiment as in Figure 4). A similar procedure of dimensionality reduction for visualizing the organization of the ensemble responses has been previously described [27]. Note that an intrinsic trial-to-trial variability was revealed since the members within one cluster presented different (although grouped) values for the two first principal components. Further, the small overlap among the different groups indicated that a good cluster separation could be reached by using linear filters, thus supporting the use of LDA classifiers for decoding the ensemble responses.

In agreement with the stimulus-related information quantification obtained at the single cell level (Figure S2A), the percentage of correct estimates yielded by the LDA classifiers grew as longer time windows for spike counting were used (panel B), with the difference across stimulus frequencies being statistically significant from a time window of 10 ms onwards (low frequency stimulation, n=9 animals, 45.6±5.3 neurons per animal; higher frequencies, n=6 animals, 48.2±6.9 neurons per animal; permutation test, all $p<0.01$). For stimulus frequencies up to 7 Hz, the decoding performance showed similar dynamics presenting a plateau from 30 ms to 100 ms after whisker stimulation. At 50 ms post-stimulus the performance was average for the low frequency (86.7±2.9% correct estimates), highest for the 1 Hz frequency (95.1±1.1%) and lowest for the 10 Hz frequency stimulation (75.4±4.7%). For the following sets of analysis we therefore maintained (as for the analysis performed on individual neurons) a 50 ms time window after whisker stimulation

After the window length was set to 50 ms, we varied the bin size used to generate the spike pattern arrays (panel C; see also Figures 1D & 4B). The differences across frequencies were highly significant at all bin sizes (permutation test, all $p<0.001$). At low frequency stimulation, the decoding performance increased as the bin size used for spike pattern formation decreased, reaching a maximum value of 93.8±1.1% at 5 ms bin size. However, the decoding performance decreased for bin sizes smaller than 5 ms. Comparable results were obtained for 1–2 Hz stimulation frequencies. Interestingly, at higher stimulation frequencies (4–10 Hz) the decoding performance decreased continuously as the temporal resolution of the spike patterns increased. For instance, at 10 Hz stimulus frequency the performance decreased from 75.4±4.7% at 50 ms bin size to 63.3±5.2% at 5 ms. This result suggests that at higher stimulation frequencies a rate code is more efficient than a temporal code, thus supporting previous reports demonstrating a transformation from temporal to rate code in the

barrel cortex at physiologically relevant stimulation frequencies (i.e. 4–10 Hz) [25,26]. As a consequence, the present results based on LDA classifiers are in contrast to the ones obtained using mutual information (see Figure S2B), a difference derived from the particular characteristics of both methods. Thus, while mutual information always increases at higher levels of pattern temporal resolution (which provide higher discrimination power), the LDA classifiers show lower performance – since creating spike arrays with larger numbers of elements derive in ‘more sparse’ and variable arrays from which no stereotypy can be extracted. Further, this result confirms 5 ms as an optimal bin size for spike pattern generation at low frequency stimulation, being in agreement with the values obtained in previous publications [3,11,21].
