# Supplementary material for: High Stimulus-Related Information in Barrel Cortex Inhibitory Interneurons
Source: PLoS Comput Biol. 2015 Jun 22;11(6):e1004121. doi: 10.1371/journal.pcbi.1004121 (PMC4476555; doi:10.1371/journal.pcbi.1004121)
Supplement: S6 Fig — (PDF) [file pcbi.1004121.s007.pdf]

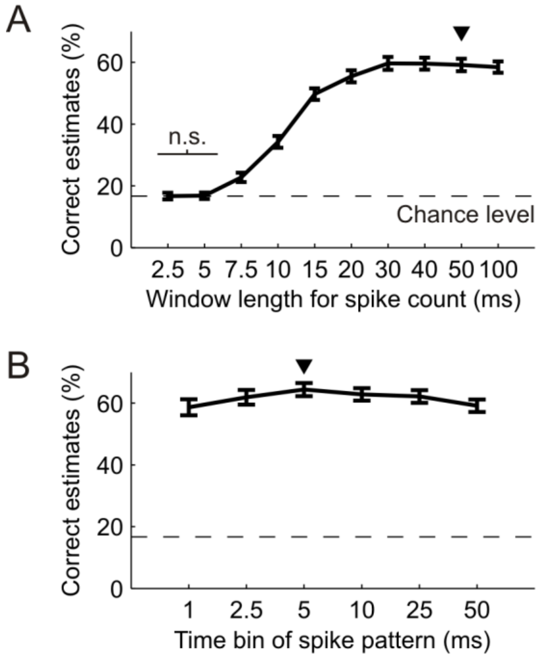

Figure S6. Effect of the window length and bin size used to generate spike counts and patterns on the resulting stimulus frequency decoding performance. (A) Effect of the total window length used to generate spike counts on the resulting stimulus frequency decoding performance. Data represent mean±SEM values averaged using all available datasets (n=16 datasets from 8 animals, see below). The values were not significantly different from chance when the window length was 5 ms or lower. Black downward triangle indicates the window length selected for further analysis (50 ms). Dashed line marks mean chance level of all datasets (16.7%, n=16 datasets). **(B)** Given a window length of 50 ms, effect of the bin size used to generate spike patterns on the decoding performance. Differences between the sample values and chance level were significant at all tested bin sizes.

The time course of the decoding performance for stimulus frequency was generally similar to that observed for stimulus location (Figure S5). The percent of correct estimates was highest when the window for spike counting was 30 ms long (59.7±2.1%), being significantly different than chance level (16.7%) from a time window of 7.5 ms onwards (paired signed rank test, all $p<0.001$), and presenting a plateau from 30 to 100 ms (similarly to stimulus location decoding performance at low frequency stimulation, see Figure S5B). Further, decreasing the bin size down to 5 ms increased the decoding performance (maximum peak 64.4±2.1%), however degrading the performance at smaller values (58.6±2.6% at 1 ms bin size). Taking into account these results, which were consistent with those obtained for stimulus location, we selected a 50 ms window length and 5 ms bin size for spike pattern generation.
