# Supplementary material for: High Stimulus-Related Information in Barrel Cortex Inhibitory Interneurons
Source: PLoS Comput Biol. 2015 Jun 22;11(6):e1004121. doi: 10.1371/journal.pcbi.1004121 (PMC4476555; doi:10.1371/journal.pcbi.1004121)
Supplement: S7 Fig — (PDF) [file pcbi.1004121.s008.pdf]

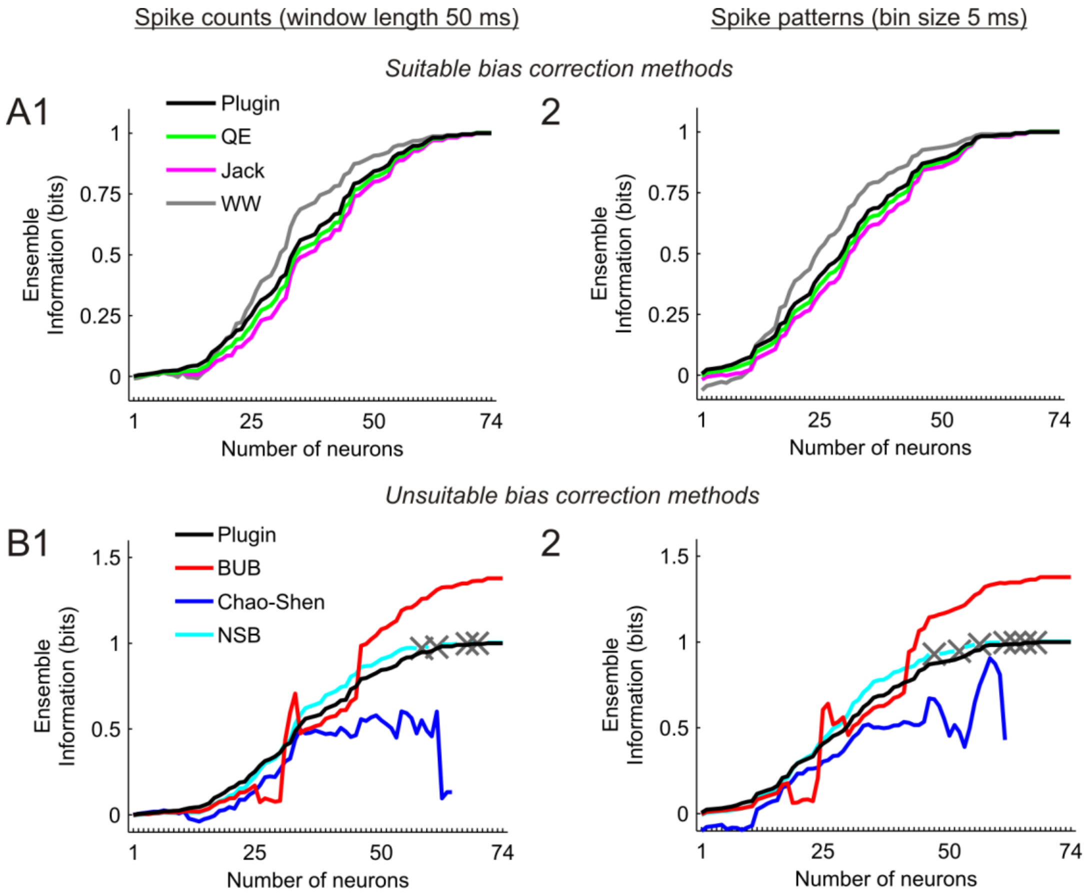

Figure S7. Effect of the number of elements representing neural responses on bias corrected mutual information values. Data included in this figure are from the same experiment analyzed in Figure 5 of the main text (n=74 neurons). Panel A presents three suitable bias correction methods: quadratic extrapolation (QE), jackknife (Jack) and Wolpert-Wolf (WW) [75–77]. Panel B presents four unsuitable methods: best upper bound (BUB), Chao-Shen and Nemenman-Shafee-Bialek (NSB) [80–82]. **(A1)** Progression of mutual information values as new neurons are added to the ensemble in ascending order, i.e. adding in each step the less informative neuron. Mutual information values were here quantified by counting spikes in a window of 50 ms. Thus, adding a neuron to the ensemble results in the addition of one element to the vectors representing population responses (of size $N_{neurons}$). When using the plugin method, the amount of information increased and reached a saturation point shortly before all neurons were included in the population. The WW method delivered higher values of information than the plugin method when response vectors contained more than ~20 elements. The QE and jackknife methods delivered slightly lower values than the plugin method, however reaching the saturation point at a similar network size. Note that the jackknife method has been shown to be a linear approximation of the bootstrap [76]. **(A2)** Performance of the suitable bias correction methods when spike patterns were used for quantification (50 ms window length, 5 ms bin). In this case the line corresponding to the QE method (green) is identical to that plotted in Figure 5B1. The resulting progression of the mutual information values was very similar to the one obtained by using spike counts;

however, in this case the maximum information value was reached using a lower number of neurons. As in panel A1, the QE and jackknife methods delivered the most accurate results. **(B)** Performance of three unsuitable bias correction methods. These three methods delivered incorrect results when responses were quantified by both spike counts (B1) and spike patterns (B2). The BUB method yielded values above the theoretical maximum mutual information when more than 40 neurons were included into the ensemble. The NSB method presented values above the theoretical maximum (or negative ones) when specific neural ensembles were quantified (marked with a gray x symbol). The Chao-Shen method presented lower information values when larger neural populations were employed; however, this progression did not match the decoding performance obtained from the LDA classifiers, which always presented a supralinear growth when neurons were added in ascending order (Figure 5A1). Similar effects to these here presented (obtained from the illustrative example in Figure 5) were observed in all 9 experiments containing more than 21 neurons (i.e. those performed with the 8x16 probe, see Methods).

When neurons were selected in descending order (i.e. adding in each step the most informative neuron), we obtained in general compatible results (data not shown). From the suitable correction methods, the QE and jackknife methods delivered again the most accurate results. The inaccuracies found in the BUB, Chao-Shen and NSB methods occurred when ensembles containing few informative neurons were considered (from ~5 neurons onwards). Thus, large response spaces were not necessary in order to obtain erroneous information values [73,79]. In conclusion, these three bias correction methods were discarded due to the inaccurate values delivered within the different analysis configurations.

In the above analyses we present values for stimulus location information, related to low frequency stimulation (<1 Hz). However, the main results and conclusions also apply to stimulus location at higher stimulation frequencies, and stimulus frequency information, which presented similar effects (data not shown).

Further, in the present analyses we consider the information related to ensembles including neurons of both types (INH and EXC) (i.e. pooled together). However, when using specific neuronal subgroups (only INH or only EXC neurons), similar results were observed, indicating that the documented effects were independent of the specific neuronal populations studied (data not shown).
