# Supplementary material for: High Stimulus-Related Information in Barrel Cortex Inhibitory Interneurons
Source: PLoS Comput Biol. 2015 Jun 22;11(6):e1004121. doi: 10.1371/journal.pcbi.1004121 (PMC4476555; doi:10.1371/journal.pcbi.1004121)
Supplement: S8 Fig — (PDF) [file pcbi.1004121.s009.pdf]

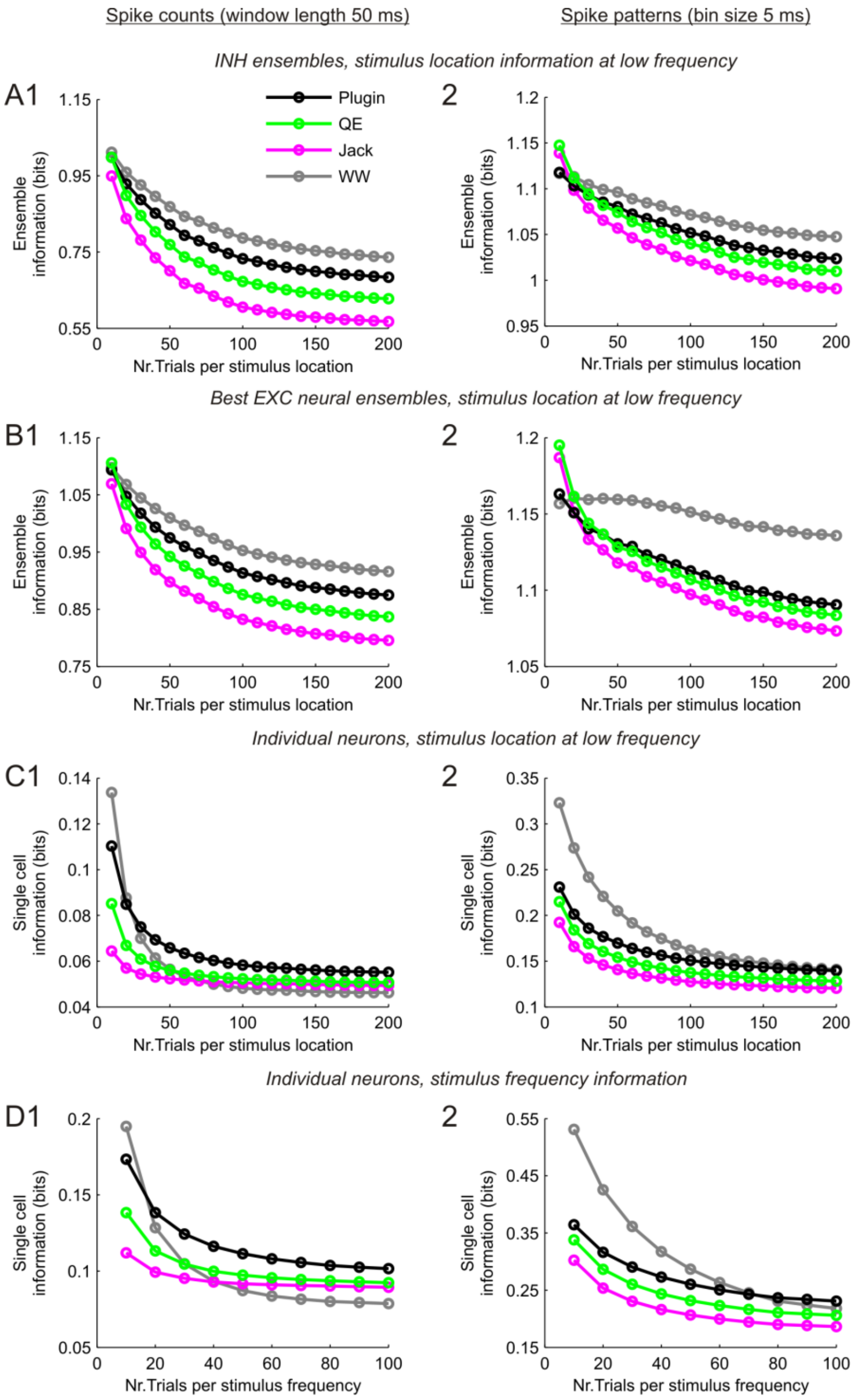

Figure S8. Effect of the number of trials used to compute bias corrected mutual information values. In each panel, mean values of mutual information computed using increasingly higher numbers trials per stimulus class. Mean mutual information values were computed using the direct method (plugin) and three suitable bias correction methods: quadratic extrapolation (QE), jackknife (Jack) and Wolpert-Wolf (WW) (see legend in panel A1) [75–77]. For each panel row, panels at the left side represent the information quantified by counting spikes in a window of 50 ms. Panels at the right side represent information values

when spike patterns were used for quantification. The first three panel rows (A–C) represent stimulus location information when the whiskers were stimulated at low frequency. The last panel row represents stimulus frequency information. **(A)** Mutual information values obtained for groups of INH neurons (5.3±0.74 neurons per animal, values averaged across animals, n=9). **(B)** Values obtained for comparable groups containing the best EXC neurons (8±2.02 neurons per animal), i.e. conveying at least as much information (1.01±0.12 bits) as the groups of INH neurons (values averaged across animals, n=9). **(C)** Mean information values conveyed by the smallest possible networks, i.e. individual neurons. No separation was done between different neuronal types; thus, values were averaged across animals using all the neurons within the populations (n=410 neurons from 9 animals). **(D)** Values of stimulus frequency related information in individual neurons (n=353 neurons from 8 animals). Note the lower number of trials in the x-axis as compared to previous panels (see Methods for a description of the number of trials in each stimulus class). Otherwise same as panel C.

For all stimulus modalities and neuronal ensemble subsets, an asymptotic decay in the values of mutual information was obtained, which progressed in parallel for the three suitable bias correction methods employed. The asymptotic decay was faster (i.e. approached asymptotic values with lower numbers of trials) in general (a) when a lower number of neurons were included into the evaluated ensemble, or (b) when the time bins used for quantifying the responses were larger. Thus and as a consequence, the number of elements representing the ensemble responses (ultimately determining the size of the total space of possible responses) was the major factor influencing the progression of the mutual information decay.

Nevertheless, when considering the ensemble sizes of major focus in our study (individual neurons, and INH and best EXC cell groups) the information decay was similar as described in previous publications in which asymptotic bias correction methods have been applied [73,79]. Further, the comparable results obtained when quantifying both the stimulus location and the stimulus frequency related information suggested an independence of this feature on the specific stimulus modality studied. In all, these data strongly support the use of our selected bias correction method (QE), and the validity of the mutual information values reported – which correctly approximated the true values that would have been obtained if considerably larger numbers of trials would have been recorded (out of reach in a single recording session).
